# Spirit of the Foreign Periodicals

**Published:** 1835-07-01

**Authors:** 


### Spirit of the Foreign Periodicals, &c.

### Abstract of M. Bouillaud's Clinical Course at the La Charité Hospital, during 1834.

During the five months, beginning with April, for which the clinical course lasted, 260 new patients were admitted into the Professor's wards. Of these, 191 were males, and 69 females. The number of acute cases was much greater than that of chronic ones; in the proportion of 200 to 60. There were but few cases of cerebral disease. Two of cerebral congestion, each of them treated with a large bleeding from the arm, with leeches applied behind the ears, cold vinegar lotions to the head, and sinapisms to the feet, were cured in from five to seven days. Two men and one woman presented the symptoms of acute inflammation of the brain and its meninges, complicated with some signs of gastro-enteritis. The treatment adopted was as follows.

*Case* 1. A man. Bled thrice; 12 ozs. each time—forty four leeches to the temples—cupped twice over the region of the heart—two blisters to the thighs—ice and the affusion of cold water on the head—enemas containing a portion of musk—died on the 10th day.

*Case* 2. A man. Bled twice; 8 ozs. each time—twenty leeches to the temples—cupped twice over the region of the heart—two camphorated blisters—cold lotion to the head—musk enemas—cured in 12 days.

*Case* 3. A woman. Four applications of twenty-five leeches behind the ears—sixteen leeches over the region of the cæcum—sinapisms to the feet—ice and cold water applied to the head—died on the 18th day.

The dissection of the two fatal cases revealed in each a highly congested state of the cerebral membranes, and a deposition of serosity in the meshes (dans les mailles) of the pia mater. The substance of the brain to a greater or less extent was of a red colour; its grey portion was softened. Such are the usual post-mortem appearances after phrenitis. The lesions found in the abdominal cavity were by no means very serious in either case. The intestinal complication was therefore merely accidental, and could not be justly considered the cause, (as it certainly is in numerous examples of typhoid fever, or follicular enteritis,) of the cerebral affection.

Some time ago, there was a case of cerebritis under the care of M. Bouillaud, which terminated in paralysis of the face, and of the inferior extremities. On dissection, the upper extremity of the spinal marrow was found in a state of ramollissement.

The next set of cases which we shall review, are those, in which the respiratory passages, or the pharynx were the seat of disease.

Four men and two women affected with tonsillitis were admitted. One local bleeding, and the application of a few leeches to the throat, were sufficient for the cure in every case. In one case, an emetic of ipecacuan was administered; but apparently without any marked effect on the duration of the malady. Fourteen cases of bronchitis, seven in persons of either sex, were speedily cured by the mere employment of soothing emollient remedies. Two cases only required general or local bleeding.

Out of twenty-six cases of pleuro-pneumonia, only three occurred in females. One half of the cases were admitted in April; and only three during the months of July and August. The duration of the disease at the time of the patient's admission, was very different in different cases: thus in one, it had existed only two days; in seven, three days; in nine, four days; in one, five days; in two, six days; in three, seven days; in one, eight days; and in two, nine days. From the third to the fourth day appears therefore to have been most frequently the date, at which the patients applied for relief. The stage of the disease, at the period of admission was equally variable. In five cases, the disease was in the first stage, that of inflammatory congestion; in seven it was passing from the first to the second, that of the red induration, or hepatization; in nine, the second stage was fairly confirmed; in three, the transition from the second to the third, that of suppuration, or purulent infiltration, had commenced, and in two, the third stage was quite established.

Of the 26 cases, in thirteen the inflammation occupied the right lung; in seven, the left; and in six, both lungs. Seven times it was confined to the inferior lobes; twice, to the superior lobes; and eleven times, the entire substance of one lung was involved.* Of the six cases of double pneumonia, in four the inflammation was seated in the bases of the two lungs; once in the summits; and in the remaining case, the entire lung of one side, and the base of the other were affected.

We come now to speak of the treatment which was pursued in these cases of pneumonia; and first with regard to general bleeding. The number of times, that venæsection was ordered in any one case, varied from nine to two. The average number was four, or five. In almost every case, copious leeching was employed simultaneously; sometimes as many as 50, or 60, at other times not more than 20 leeches were applied over the seat of the greatest pain, or directly over the region of the heart.

In the majority of the cases, cupping also was resorted to. The rest of the treatment consisted in the occasional use of blisters, and in the administration of croton oil as an active purgative.

---

* These data agree in the main, with the conclusions which M. Chomel in the Art. Pneumonie, Dict. de Medecine, announces to be the results of his enquiries. Morgagni has stated that pneumonia occurs more frequently on the right, than on the left side. Of 27 fatal cases examined by Chomel in the Winter 1812-13, in thirteen, the right lung only was found affected; in seven, the left one only; and in the remaining nine, the inflammation had attacked both lungs, the right one however being generally more seriously injured than the left one. M. Andral's conclusions coincide with those of Chomel and of Bouillaud. He is of opinion that pneumonia on the right is, at least, as common again as the disease on the left side.

With respect to the comparative frequency of the inflammation in different portions of the lungs, the following are the results of fifty-nine fatal cases examined after death by M. Chomel. In 13, the inflammation occupied the summit of the lung; in 11, the base; in 31, the entire lung; in three the posterior edges of both lungs; and in one, the middle portions of both lungs. From these, facts it seems that inflammation more frequently attacks the summits, than the bases of the lungs; but on this subject M. Chomel observes, that, independently of the too small number of his observations to warrant any decided conclusions to be drawn from them, if inflammation of the upper lobes is more frequently fatal than that of the lower lobes, we cannot judge very accurately of the relative frequency of the two sets of cases, merely by post-mortem examinations. M. Andral has collected the reports of 88 cases, some of which proved fatal, but the greater number terminated successfully; and of these, the inflammation appeared to occupy the lower lobes, 47 times; the upper lobes, 30 times; and the entire substance of one lung, 11 times.

The attentive reader will doubtless compare these results respecting pneumonia, with the results of Louis' researches on the relative frequency of tubercular disease on the two sides of the chest, and in different portions of the lungs.

Two only of the 26 cases proved fatal. The average duration of the treatment was about 8 days, the longest period being 14, and the shortest 4 days.* During the latter months of the clinique, M. Bouillaud gave a decided preference to cupping, as a means of local depletion in pneumonia, over leeching. The impression thus made on the disease is more speedy and decided; and, moreover, the exact quantity of blood drawn can be more easily ascertained.

Simple *pleurisies* were less frequent than inflammations of the lungs themselves. There were only four cases admitted during this clinique; three of these occurred in men, and one in a female. In two of the cases, the pleurisy was simple and uncomplicated—in one, it was associated with pericarditis; and, in another, with acute rheumatism. These cases of pleurisy did not require such active depletion as the cases of pneumonia. The application of a blister once, or oftener, was practised in all the cases. Independently of these four pleuritic patients, there was a fifth, who had been admitted before the commencement of the clinical course, and in whom a fatal pleurisy was induced by rupture of a pulmonary tuberculous excavation.

The number of cases of *acute rheumatism*, or, as it ought rather to be called, of *arthritis*, admitted during the five months, from April to September, was sixteen. Eleven were in men, and five in women. One case only occurred in April, five in May, three in June, five in July, and two in August. In all the cases, the articular inflammation having appeared at first in one joint only, affected, in its progress, almost every other joint of the body. To the treatment of acute rheumatism by vigorous antiphlogistic remedies, it has been objected that, whenever they have effected a speedy cure, the disease had already existed for a considerable time. It is, therefore, important to determine its duration, in each of our sixteen cases, at the date of their respective admissions into the hospital. In three, it had existed for 3 days—in three, for 5 days—in six, for from 6 to 8 days, and in four for 15 days. The treatment consisted in vigorous depletions of blood, general as well as local. The largest quantity of blood drawn, in any case, was 94 ozs.—about 14 ozs. at seven different times; the smallest quantity was 14 ozs. drawn at one bleeding. The quantity usually drawn at a time was between three and four palettes (a measure holding four ozs). The number of leeches applied, in any one case, varied from 157 to 12; and the frequency of cupping from once to three times. The remainder of the treatment consisted in blistering, compression combined with mercurial inunctions, the endermic use of morphia and baths, as external remedies, and in the internal exhibition of opium, morphia, Dover's powder, and occasional purgatives. The duration of the treatment, after admission into the hospital, varied from 7 to about 16 days; in one case, where the affection was limited to the two wrists, it was continued during 23 days; and in another case, where the disease was in a subacute form, and confined to one knee only, it lasted for 17 days. It is worthy of notice that, in the latter of these two cases, no general bleeding was practised, and the treatment consisted in cupping three times, and in employing compression along with mercurial frictions; and that, in the former, the patient, in whom the disease was confined to the two wrists, was bled six times—in all, to the amount of 80 ozs. In conclusion, it may be stated, the average quantity of blood drawn was about 42 ozs. at three separate operations, besides 6 or 8 ozs. by the cupping-glasses, and the discharge from between 20 and 30 leech-bites. The average duration of the treatment in the hospital was 11 days; and of the disease, from its commencement, about 19 days. (The English physician will, no doubt, be surprised to find that, in none of the cases of

* This announcement, the truth of which cannot be gainsayed, exposes the error of those who state that recovery is always tedious, after " un traitement energiquement antiphlogistique."

acute rheumatism alluded to in this report, was any form of mercury or of colchicum employed. Both are remedies of great power. The almost immediate cessation of the most severe articular pains on the supervention of salivation, must be well known to every practical man.—REV.) There is a circumstance in the history of acute rheumatism, which, of late years, has attracted much the notice of physicians—we mean, the very frequent co-existence " d'un trouble du coté du cœur." This complication consists sometimes in well-marked (franche) pericarditis, or in an endo-carditis (inflammation of the serous investiture of the cavities of the heart), and, at other times, in an increase of the enlargement and induration of the valves (the consequences of a former attack of endo-carditis), or in the presence of organized coagula within the ventricles or auricles. As to the predisposing cause of these cardiac lesions in acute rheumatism, we believe it may justly be traced to the analogy of structure which exists between the serous membranes of the heart and the synovial membranes of the joints. According to the apt expression of M. Bouillaud, the heart, enveloped in the pericardium, may in some respects be viewed as a " veritable articulation; car on trouve dans ce systeme d'organes des mouvemens, des surfaces qui glissent l'une sur l'autre, une membrane sereuse, et un tissu fibreux qui la double."

Twelve cases of *acute erysipelas*, six in persons of either sex, were admitted. In nine, the disease occupied the face, and, in the remaining three, the superior extremities. Phlyctenæ appeared in a few of the cases. In all, the disease was strongly marked, and all of them yielded to an active antiphlogistic treatment. One of the men lost 72 ozs. of blood by two bleedings from the arm; and several of the other patients were bled three or four times, usually to the amount of from 12 to 16 ozs. at each operation. The application of large numbers of leeches to the inflamed parts was of signal utility. The treatment lasted for from 4 to 14 days, the average period being six or seven.

Among the cases of exanthemetic disease, there were eleven of *small-pox*. (It appears that small-pox was prevalent in Paris during the Spring and Summer of last year.)

Of these eleven cases, four occurred in persons who had been vaccinated, and of these four cases, one was of the severe confluent form of the disease. In almost all the cases, the eruption was very copious, and in four it was confluent. Three of the confluent cases of the disease occurring in unvaccinated patients, proved fatal. In one-half of the cases, the treatment consisted in the mere use of gentle emollients—in the other half, the detraction of blood, either at the commencement or during the progress of the disease, at the setting in of the secondary fever, was necessary. Thus, when the eruptive fever was very intense, and the eruption promised to be very copious, a bleeding of from 12 to 16 ozs. was practised; and, whenever there seemed to be any tendency to congestion within the head, a number of leeches were applied behind the ears. " I cannot," says M. Pelletan, the reporter, and he doubtless expresses the sentiments of his preceptor, Professor Bouillaud, " now boast, as I have hitherto done, of the utility of the antiphlogistic treatment in small-pox; there is a something else, besides mere inflammation, present in this disease. This *something* is of a specific nature. The blood has become altered in its qualities; for, if we examine it after it has been drawn from the arm, it will be found to be, not firm and dense, as in genuine inflammations, but soft, diffluent, and having all the characters which it usually presents in typhoid enteritis." The inhalation of chloruretted vapour was found to be a very useful remedy in most of the cases—it tended to cleanse the throat and mouth more effectually than any other means.

In nine cases of measles, six of which occurred in males and three in females, the accompanying bronchitic affection was invariably present to a greater or less degree. In two it was extremely severe, and both patients died asphyxiated. In one case, the rubeolous rash was complicated with miliaria, and, in another, with scarlatina, and with cynanche tonsillaris. The danger of measles is always proportionate to the severity of the accompanying bronchitic affection; but let it not be forgotten that the antiphlogistic treatment, although the only safe practice in such cases, has not the same control over the pulmonic complication of rubeola (which, and such like varieties of the disease, might be appropriately called "peripneumonia notha," instead of the subacute form occurring in old people), that it exerts over the open and idiopathic inflammations of the lungs.

[This is an important concession on the part of a Broussaisist. We may soon expect a considerable modification of the original tenets of this talented but one-sided physician.—Rev.]

Three cases of sciatic neuralgia were cured by the acetate of morphia, used endermically.

Five cases of ague were speedily cured. In one case, the febrile paroxysm was arrested by the application of the sulphate of quinine to the denuded cutis; and in another by digitalis, used in the same method, and likewise taken internally at the same time.

Three cases of lead colic yielded speedily to the employment of croton oil and of opiates.

Three cases of chorea, two in females and one in a male, were successfully treated with etherial potions, pills of valerian, and lavements, containing musk, camphor, and assafœtida.

The cases of "embarras gastrique," or of slight gastritis, amounting in all to twenty, did not present any remarkable phenomena. Seven of the cases were treated with one or more emetics of ipecacuan; but these cases did not recover more quickly than ten in which a mere emollient treatment was pursued. In the remaining three cases, the symptoms being rather more urgent required the application of leeches to the epigastrium.

Under the term of gastro-duodenitis, M. Bouillaud arranges that form of typhoid fever which exhibits a bilious type or character, and which is denoted by pyrexia, accompanied with sickness, bilious vomitings, great depression, an icteric discoloration of the skin, and a sense of weight and distress in the epigastric and hypogastric regions. This form of fever is not so dangerous as that whose pathological seat is in the mucous glands of the intestines, and which is so well known, in the present day, by the names of follicular enteritis, entero-mesenteritis typhoidea, dothinenteritis, &c.

Fourteen cases of the genuine gastro-enteritis were admitted, and they were all successfully treated. In nine, venæsection to the amount of 14 or 16 ozs. was deemed necessary, once or oftener; and local depletion of blood, by leeches or cupping, was very generally combined with it. On the whole, however, this form of disease does not require a very active depletory treatment. It was remarked that three of the cases, in which emetics and purgatives were employed, were of more tedious recovery than the rest.

Of the follicular enteritis, no fewer than 32 cases were received into the clinical wards. Of this number, nine only occurred in females. All the patients, with the exception of two, were young—their ages being from 17 to 25. The two oldest were only 37 years old. With respect to the treatment pursued, we may state that, on the average, each case required two general bleedings, the application of 28 leeches to the abdomen, and one cupping. To these means were added the occasional use of blisters, sinapisms, and chloruretted baths. Five out of the thirty-one cases proved fatal.

It is to be particularly noticed, in estimating this proportion of mortality in Bouillaud's practice, that, contrary to the custom of many physicians, he does not reckon among the proper typhoid fevers, those cases which we discussed in the preceding section, viz. the gastro-duodenites or bilious fevers, and of which not a single example proved fatal.

An abridged report of one or two of the cases of the follicular enteritis, will better explain M. Bouillaud's practice than any general description.

*Case* 1. This patient was admitted on the 8th day from the commencement of his illness. The symptoms were, extreme debility and exhaustion, tendency to stupor,—the lips, teeth, and tongue were parched, the latter was covered with a yellowish coating in the middle, the edges being red—the skin was dry and burning—pulse 84—intense headache, and tinnitus aurium—extreme thirst—abdomen tender and tympanitic: a gurgling noise heard, when the ileo-cæcal region was pressed—diarrhœa, of several days' standing, troublesome, but not so severe as it had been; sleeplessness, and tendency to delirium.

After he had been bled, the heat of the skin abated considerably, and there was less headache and prostration of strength. Thirty leeches were now applied on the abdomen. The tongue became moist, and rather cleaner; the saliva, which hitherto had been acid, no longer reddened litmus paper. On the following day, twenty more leeches were applied on the abdomen, and these had the effect of removing the tympanitic distention, the gurgling sound, and the looseness of the bowels. In the course of a few days, this patient was quite convalescent.

[*Remarks*. This case appears to us to have been one of mild typhus, the typhus mitior of Cullen; and certainly did not exhibit the "extreme gravité" assigned to it by the French reporter. The symptoms scarcely warrant us in affirming that there was any inflammatory affection of the intestinal mucous glands.—REV.]

*Case* 2. The patient was admitted on the sixth day of the disease. In addition to the symptoms which were present in the former case, there was a most extensive bronchitic affection; on ths right side there was an incipient pneumonia, indicated by a distinct crepitant râle on auscultation, and by the peculiar, viscid, rust-coloured sputa. The lungs and the intestines were, therefore, very seriously affected in this case; and, if we add that the patient's constitution was soft and lymphatic, that his character was "d'une pusillanimité honteuse," and that the little intelligence he possessed was uniformly employed only "à nous tromper, pour satisfaire sa gourmandise et sa voracité," the reader may form some idea of the difficulty which was experienced in the treatment. The first and most urgent indication was to relieve the pulmonic congestion; else the dyspnœa might have soon become suffocative. The patient was, therefore bled three times, to the amount, in all, of 44 ozs. in the first 24 hours, and about 8 ozs. of blood were taken by cupping also. This active treatment so effectually quieted the pulmonary symptoms, that the whole attention might now, with safety, be directed to the abdominal distress. But, in consequence of the repeated imprudences of the patient, this was not very easily effected. He became so extremely feeble, that, for several days, his life was in very imminent danger. Several eschars formed on those parts of the body on which he pressed, when lying. The use of the chlorurets, both internally and externally, was steadily persevered in, and eventually with the happiest effects. The recovery however was very tedious.

*Case* 3—we are told, was an example, "non moins grave, de cette terrible maladie." The disease had commenced eight days before her admission into the hospital, with diarrhœa and bilious vomitings. Her actual state was as follows:—Features pale, shrunk, and inanimate, exhibiting a yellowish discoloration on the alæ nasi, and the lower part of the face; mind confused, and, as it were, oppressed, so that her answers were slow and incomplete; the skin was dry and burning; pulse 120, feeble; tongue dry, red at the edges, and having a white coating in its centre; mouth parched; breath fetid; saliva acid; nausea, and occasional bilious vomitings; epigastrium distended and painful; the whole of the abdomen more or less tender, especially over the ileo-cæcal portion, where a gurgling noise was produced by pressure; severe headache, vertigo, restlessness, and stupor, alternating with occasional delirium.

As to the lungs, there was nothing very worthy of notice; the breathing was very hurried, and each act of inspiration was incomplete and jerky (saccadé). The patient was bled to the amount of ten ounces, but without any manifest change in the symptoms. Twelve leeches were then applied over the cæcum: the tympanitic distention and gurgling abated in consequence; but the general state was not affected. Twenty-four leeches were re-applied to the iliac regions; but no amendment followed. Next day, there was considerable subsultus tendinum in the shoulders, arm and face; the adynamia had become extreme; the urine was ammoniacal, and the coma was constant, except when it gave way to delirious excitement. Blisters had been applied to the calves of the legs and sinapisms to the feet; and the chloruret of soda had been used both inwardly, and as an external application to the surface of the body. The blistered surfaces had become threatened with gangrene; but this tendency was fortunately arrested by employing dressings of camphorated spirit and cinchona. Contrary to all expectation, this patient began to exhibit signs of recovery. The pyrexia abated, the diarrhœa and bilious vomiting ceased; the tongue became moist and clean, the saliva lost its acidity, the appetite returned, and the urine resumed its healthy properties.

The following case, though not strictly belonging to the present section, may be introduced here with advantage.

*Case* 4. *Intense choleriform peritonitis, induced by perforation of the intestines.* A woman 58 years of age was sent into the hospital from the country, as a genuine cholera patient. The symptoms were, utter prostration; the eyes sunk and glazed; the arms and hands icy-cold, and bedewed with a moisture; the tongue cold, and of a purplish-colour; the pulse quite imperceptible; frequent efforts to vomit, the rejected matter consisting of green bile mixed with flocculi; the abdomen hard, distended, and tender on the slightest pressure, On the evening previous to her admission, this woman had been suddenly seized with violent colicky pains, during the continuance of which, she had, at three different times, voided some blood per anum, also with vomiting, difficult enuresis, &c. She stated that for some time past she had been subject to dyspepsia, frequent recurrence of vomitings, and excessive constipation, and to a gradual loss of all her strength and energies. The symptoms of her present attack became more and more alarming, and she died during the first night she was in the hospital.

Considering the extreme rapidity of the disease, the patient's previous state of health, viz. the obstinate constipation, and the bloody stools following an attack of severe colic, and coupling these facts with the present existing symptoms, which denoted an intense peritonitis, M. Bouillaud suspected that the contents of the intestines had become effused into the abdominal cavity; a suspicion which was amply confirmed by the post-mortem examination. Besides the marks of an intense peritonitis, the mucous coat of the intestines was found covered with myriads of insulated minute follicles.

The whole extent of the large intestine was loaded with indurated fæcal matter, and on tracing it to the rectum, a large opening was found in that portion of the gut which is in contact with the uterus. This opening was as big as a five-franc piece, and the edges were livid and gangrenous. A quantity of the fæces had escaped into the cavity of the pelvis. The other lesions do not require particular notice.

This case is instructive, as exemplifying an error, into which medical men are too apt to fall; viz. that of attributing, during the existence of any epidemic, every case whose symptoms are analogous to those of the prevailing disorder, to one exclusive cause.

Having now mentioned all the cases of acute disease, which were admitted during the clinical course, it only remains to allude to the *chronic* cases. Tubercular phthisis and organic affections of the heart were perhaps the most numerous of these. The treatment of such cases, as were not manifestly incurable, consisted in occasional ocal bleedings, and the internal use of digitalis. Some cases of chronic inflammation of the stomach and bowels, illustrated the benefits of the endermic employment of morphia, and of mercurial inunctions. One case of ascites, which had followed an obstinate chronic peritonitis, was much relieved by the rubbing in of the ung. hydrargyri over the whole abdomen; and another, which was dependent on enlargement of the spleen, was benefited by the use of diuretics after the operation of paracentesis.—*Journal Hebdomadaire.*

---

### M. Louis on the Effects of Blood-letting in Pneumonia, &c.

The therapeutic effects of sanguineous depletion, have been alternately praised and disparaged, to an extravagant and unjust degree, merely according to the doctrinal views of physicians, and with far too little regard to the specialities of the prevailing disease, or of the individual cases. Of late years, the subject has been studied with more than usual care. M. Louis, whose character we have already exposed in the present number of our Review, has for some years past been endeavouring to submit to numerical, or arithmetical accuracy, the results of blood-letting in various inflammatory disorders, and more particularly in pneumonia. His earliest paper on this subject appeared in the Archives Generales de Medecine for 1828.

The bleedings which M. Louis employed in most of his cases, were, as we shall presently see, small, but frequently repeated; and the results of the remedy so practised were certainly far from being encouraging. The proportion of deaths was nearly one in every three cases. Not discouraged however by want of success, he has diligently continued his enquiries, respecting the curative effects of bleedings in small quantities,* in pneumonia, cynanche, erysipelas, &c. and he has recently published an account of his experiments, in a separate brochure, entitled " Recherches sur les Effets de la Saignée dans quelques Maladies Inflammatoires, &c." The first chapter of this memoir is a reprint of his former paper, and contains the reports of 78 cases of pleuro-pneumonia.

Of these 28 proved fatal; or rather more than one in every three cases. All the patients were treated by bleedings, " pratiquées comme on le fait ordinairement;" that is, in small quantities, generally of from 10 to 15 ounces. One patient only was bled so often as seven times.

| | |
|---|---|
| Three patients were bled | 5 times. |
| Twelve - - - - - - | 4 —— |
| Sixteen - - - - - - | 3 —— |
| Thirty-four - - - - | 2 —— |
| Ten - - - - - - - - | 1 —— |

Thus it appears that the number of those who were bled only once, and twice, exceeds very considerably, the aggregate of the other cases, in which venæsection was practised more frequently. We shall now briefly state the immediate results of these bleedings. The duration of the pneumonia appears not to have been much influenced by them. When the patient was bled before the fourth day of the disease, it usually lasted 17 days; when not bled, until or after this date, the usual duration was about three weeks. The pain was in no case suddenly arrested (jugulée) by the venæsection; but the speediness of its cessation was always in proportion to the earliness of the practice. Thus it usually continued for

---

* The French reviewer adds; " we say, *in small quantities;* for it is well known that Professor Bouillaud, struck with the extraordinarily unpropitious results of blood-letting, as practised by M. Louis, and by many of the other Parisian physicians, was induced to ask the question, whether by changing the *method,* without changing the *means,* we might not hope to be more successful. He has most satisfactorily resolved this most interesting medical problem, and has proved by the most unanswerable argument, the simple deduction from a multitude of facts, the immense superiority of blood-letting " à haute dose."

about six days, when the patients had been bled during the first four days of the disease, and rather longer than eight days, when the bleeding had been delayed beyond this period. Some of the other symptoms, especially the characters of the sputa, were similarly influenced, according to the promptitude or tardiness of the treatment. The "crachats, visqueux, rouillés, ou marmalade d'abricots," continued for five days, in those patients who had been bled during the first three days, and for six or more days, if the bleeding had been delayed. The characters of the sputa became more marked and distinct after bleeding, in the greater number of those cases where it was employed near the commencement of the disease ; and, on the contrary, they were less apparent on the day after the operation, in the cases where it had been delayed. M. Louis very justly attributes these differences to the circumstance of the disease being farther from, or nearer to, its term or issue, in the two sets of cases. Similar remarks may be made as to the effects of bloodletting, employed at different periods of the disease, on the auscultatory signs of the disease, such as the crepitation, the resonance of the voice, the œgophony, the dulness on percussion ; and also on the state of the pulse, and the heat of the surface.

Sometimes all these symptoms became very much mitigated, soon after the blood was drawn, if the operation had been performed "au debut" of the pneumonia.

So much for the effects of the remedy in this disease. In the cases of erysipelas of the face, recorded by M. Louis, the influence of sanguineous depletion, either on the duration or the event of the disease, appears to have been very feeble indeed. Bloodletting was employed in 21 out of 33 patients ; and the only result obtained from its adoption, was a very inconsiderable shortening of the duration of the malady. A similar remark is applicable to the cases of cynanche in which it was practised. The disease was only shortened by about one day.

The second chapter is occupied with the reports of all the new cases, or of those which have been treated by M. Louis from the year 1830 to 1833. The amount of the bleedings was, in general, larger than had been employed in the preceding cases, and the *success has been correspondingly greater*. Of 29 patients, four only died. The largest quantity of blood drawn at once was 20 ozs. This was done in three of the cases; in the others, the average quantity was about 16 ozs. at each operation. On the whole, the entire quantity was considerably greater in this, than in the former set of cases; and the more immediate results of the remedy were proportionately more satisfactory. The duration of the pneumonia, when the bleeding had been practised from the second to the fourth day of the disease, was, on the average, between fifteen and sixteen days; and, when it had been delayed until from the fourth to the seventh day, between 18 and 19 days.

The persistance of the individual symptoms, such as the pain, the peculiar expectoration, the crepitation, bronchophony, the state of the pulse, &c. was also somewhat shorter in the present series of cases. The four patients who died, had not been bled until after the fourth day from the commencement of their attacks.

Viewing the two sets of cases reported in M. Louis' memoir in connexion with each other, we cannot fail to put the question to ourselves—Whence the difference in their results ? The obvious, at least to us, conclusion seems to be, that the more vigorously and decidedly the depletory plan was pursued, the more successful was the practice. The author, however, takes a somewhat different view of the question ; and he attributes the greater success, in the latter series of cases, to the employment of the tartrate of antimony (given in from 6 to 12 grains in the course of 24 hours) ; although, according to his own admission, the disease lasted, when this medicine was employed, three days longer than in those cases where it was not used at all—an event which M. Louis explains by stating, that the exhibition of the antimonial had been delayed too late, viz. until after the fourth day of the disease. But there is surely some degree of inconsistency in these reasonings. If the duration of pneumonia was prolonged three or four days, where bleeding had not been practised until the fourth day, we need not certainly seek for any other interpretation of the tardy cure of certain cases.

At the close of this section, M. Louis alludes to the action of blisters in pneumonia. He is of opinion that they have not any beneficial influence on the disease. He has, therefore, abandoned the use of them, not only in pneumonia, but also in pleurisy and pericarditis; " car," says he, " un examen rigoureux des faits m'a toujours convaincu, que les affections inflammatoires aigues, loin de preserver de l'inflammation les organes qui n'en sont pas affectés primitivement, en sont une cause excitante." From this, it seems that M. Louis regards the effects of a blister as identical with an active inflammation. Surely this is not correct.

Such is a brief review of the leading assertions in M. Louis' memoir. The conclusions to be drawn from the whole enquiry are certainly not very encouraging, either to patient or doctor. If the mortality of pneumonia, a disease so frequent and so common, amounts to one-third of all those affected with it, medical men ought surely to renounce the ancient regime of treatment, and make an experiment, at least, of other means. Hitherto, but little progress has been made in this enquiry. The tartar-emetic, indeed, has been extravagantly eulogized by some, as almost infallible: but our author, who is decidedly, and, we may add, very properly, partial to its use, candidly admits that the cases treated chiefly by it, were quite as tardy in recovering as those where it was not administered. Nevertheless, it is a most potent and useful remedy, and is admirably adapted to such cases as are not well fitted for large depletions of blood. But we need not despair. We have in our possession a remedy which has been long known, and is continually employed, but the full value of which M. Louis, and, perhaps, the majority of French physicians, have not yet fully estimated. This remedy is no other but venæsection; but venæsection " à haute dose," and not " à petite dose, comme on le fait ordinairement." The very circumstance of a physician, so eminently distinguished for all the attributes of a good practitioner as M. Louis, having been so unsuccessful in the treatment of pneumonia, " comme on le fait ordinairement," must satisfy every one that the " methodus medendi" is faulty somewhere.

His unquestioned integrity is a sufficient voucher for the authenticity and correctness of the facts; and his practical talents for the skill with which the remedies were employed. Fortunately there are other facts, equally authentic and correct, facts which have been confirmed over and over again, and of which numerical tables, as copious and as exact as any detailed by M. Louis, have been kept; and the results of these are far more encouraging than those which we have hitherto been contemplating. Venæsection has been the instrument which has enabled us to obtain these results; but venæsection not small and imperfect, but " portée á haute dose." To Professor Bouillaud belongs the merit of having first distinctly demonstrated the efficacy of large bleedings in pneumonia. (We shall not deny the justice of this claim, in reference to our brethren in France; but its ridiculous impudence, if it is intended to apply to other countries, is too obvious to need any refutation: [be it remembered that the claim is advanced in a periodical, under the management of M. Bouillaud himself.—REV.] The average quantity of blood he draws in pneumonia, may be stated at rather more than four pounds and a half; whereas, in the practice of M. Louis, this quantity seldom exceeds two pounds and a half. The average mortality, among M. Bouillaud's patients, is not greater than in the proportion of one to eight and a half, which is considerably lower than even in the second series of M. Louis' cases.

Moreover, the medium duration of the disease, in the wards of La Charité, is from 8 to 12, and not from 18 to 20 days.

With respect to M. Louis' condemnation of blisters in pneumonia, it deserves notice, that they were rarely used, unless in cases which, " peu influencés par la saignée, laissaient des craintes sur l'issue." In the practice of M. Bouillaud, blisters have been found of very signal advantage.—*Journ. Heb.*

---

Since writing these observations, we have read an able letter on the subject of M. Louis' memoir, published by one of his former pupils, M. Donné. We shall avail ourselves of some of his remarks, as they tend to confirm the opinion which we had formed ourselves. M. D. very justly observes, that the facts and reasonings adduced by M. Louis, do not warrant us in deducing any exact conclusions as to the influence of bloodletting, in general, on the course, duration, or issue of pneumonia; because it is very evident that the only correct way to obtain such a knowledge, is to compare a certain number of cases, treated altogether without bleeding, with the same number of equally severe cases, treated with it. Now, in all M. Louis' cases, more or less blood had been withdrawn at some period of the disease. The value of M. Louis' facts appears to consist chiefly in affording data, to prove the relative advantages of an early and of a tardy recourse to the remedy; and certainly we had thought that it needed not a physician of M. Louis' talents, nor so minutely recorded tables of cases as he has published, to prove the infinite superiority of the one method of treatment over the other; but we find that we have been mistaken, at least if the results of these tables are to be received as guides of our judgment. It appears that, out of 41 cases, in which bleeding had been employed during the first four days of the disease, 18 died; and that out of 36 other cases, in which the bleeding had been delayed beyond the fourth day, only nine were fatal. Were we, therefore, to trust exclusively to the numerical method of investigation, without due regard to the circumstances of each case, we should come to the very unexpected conclusion, that it is better to delay bleeding, for some time after the mischief has been fairly established! " Il y a des gens, auxquels des colonnes de chiffres en imposent! Toute la science se reduit, pour eux, à l'addition et à la soustraction."

Without disparaging the exactness of the arithmetical mode of enquiry, we quite agree with the truth of the axiom —" perpendendæ, magis quam numerandæ sunt observationes." Certain departments of medical science are much more likely than others to be improved and advanced, by minutely-recorded registers of facts. Thus the morbid anatomy, the etiology, and what may be called the statistics of diseases, cannot be satisfactorily examined in any other method. If we wish to know on what lesions the symptoms of a disease depend, or, at least, what lesions are generally found on dissection, after the existence of a certain train of symptoms, it is obvious that we cannot hope to arrive at any accurate knowledge unless we make a multitude of observations, and have these carefully arranged. No short series of examinations can ever suffice—it may give the hint and suggest the idea, but beyond this it cannot go; and, in order to arrive at any exact conclusions, we must have series after series, each faithfully recorded and numerically arranged. The signal advantages of such a system of pathological enquiry have been beautifully illustrated by Louis, in his researches on phthisis. His shrewd and unbiassed mind soon perceived that, in describing the morbid anatomy of a disease, it was not sufficient, nay, it was not correct merely to detail, however exactly, every lesion and abnormal appearance which was revealed on the dissection of fatal cases; and for this very obvious reason—that some of these lesions, and some of these abnormal phenomena, were strictly and essentially the results of the disease in question, and that others were merely coexistent with, and not necessarily dependent upon it. For example, he assures us that, whenever he discovered any genuine tuberculous matter in the spleen, in the kidneys, in the intestines, mesenteric glands, or, indeed, in any part of the body, not including the lungs, he invariably found the same morbid deposit in these last-named viscera; intimating thereby some necessary connexion between tubercles in the lungs, and tubercles any where else.

According to his researches, also, we have been taught more correct doctrines respecting the disease which has been styled laryngeal phthisis. Until very recently, it was the generally-admitted opinion, that all the symptoms of genuine pulmonary consumption might be induced by ulcerous diseases of the larynx and trachea, while the lungs remained perfectly sound. But this opinion has been proved to be erroneous; and it is now no longer denied that, in every case of tubercular disease of the air-passages, there is a co-existent tubercular affection of the lungs themselves. Such a fact could have been proved only by comparing a host of exact observations.

As illustrative examples of a similar connexion, and of the mutual dependence of diseased actions in different parts of the body at the same time, we may allude to the fatty disorganization of the liver, and the enlarged and ulcerated state of the mucous glands of the intestines, in phthisical cases. These lesions are almost exclusively found in patients who have died of tubercular disease; and they may, therefore, be considered as the direct and legitimate effects of such a state. But there are other lesions, as frequently met with in phthisical cases as any of the lesions which we have specified, and which nevertheless, cannot be considered as pathognomonic, or specially appertaining to and induced by phthisis: we may mention, as examples, the effusion of serous fluid into the pleuræ, adhesions of these membranes, portions of hepatized lung, &c. which are so common appearances after phthisis. But then they are almost as common after other chronic and protracted illnesses as after phthisis.

The division, therefore, of the post-mortem appearances, found after a long-continued disease, into such as are special to, and characteristic of it, and into such as are common to it along with other chronic diseases, is of primary importance in the study of pathological science. Hitherto it has been far too frequently overlooked; and the errors which have in consequence arisen, in endeavouring to search out the physical causes of disease, have been most perplexingly numerous. Typhus fever furnishes an apt illustration of this remark. How various and conflicting have been the opinions as to the primary and essential morbid condition, or, as it used to be called, "proximate cause," of this very common disease; and how unstable and unsatisfactory, in consequence, has been the treatment of it! The humoral doctrine of Boerhaave, the nervous doctrine of Cullen, the cerebral doctrine of Clutterbuck, the gastro-enteric doctrine of Broussais, and, lastly, the dothinenteric doctrine of Bretonneau, Bouillaud, and others of the French school, all these doctrines have arisen from the faulty method of investigating the subject of enquiry. The authors have taken too partial and exclusive a view of the disease—they have directed their attention to one set of symptoms, or to one set of post-mortem appearances, to the omission of other symptoms, and of other appearances; their facts or data have been too insulated and disjoined, and their conclusions have been, in consequence, too contracted, and often erroneous. We have been induced to extend these remarks, as the subject is not only of great scientific interest, but of direct practical importance. Does not the therapeutic history of typhus fever shew how fickle the art of medicine too frequently has been?

The antiseptics and antacids recommended by one school, the antispasmodics and diaphoretics by another, the cordials and stimulants by a third, the application of leeches to the head, epigastrium, hypogastrium, &c. by a fourth class of physicians;—all this fluctuating uncertainty of practice ought surely to lead a philosophical mind, "nullius in verba magistri jurare," but to study the book of Nature for itself, with no fond theory to establish, and no rival doctrine to assail. The exactness of the numerical method of enquiry is admirably calculated to correct this great evil of medical pursuits. If, for example, the physicians whom we have mentioned by name, as the authors or chief advocates of certain doctrines, had prepared registers, tabularly arranged, of a multitude of typhus cases; these registers, presenting a succinct account of the mode of invasion, the progress, the duration and issue of each, with minute details of every abnormal appearance found on the dissection of those who had died, we should, long ere now, have arrived at much more accurate conclusions as to the seat, and a much more rational method of curing the disease, than has usually prevailed.

In typhus, as in every other disease, we should interrogate, not one only, but all the functions of the living body; and examine, not one only, but all the viscera and systems of the body after death. No physician has deserved better of medical science, in this respect, than M. Louis; but, while we cheerfully admit his claims to distinguished eminence, we must not disguise from our readers, that we are not prepared to follow him in some of his doctrines as to the treatment of diseases. The numerical method of enquiry, which he has endeavoured to introduce into the treatment of typhus fever, has satisfied us, that even the most cautious and scrupulous enquirer is apt to be misled into strange admissions, when he attempts to apply the rules and axioms of an exact science, like arithmetic, to the explanation of the phenomena of an ever varying object, like that of the human body, especially when the equipoise of its functions is disturbed by disease. We have already seen that one of the results of his tables is the unexpected announcement, that a larger proportion of the patients who had been bled before the fourth day, died of the disease, than of those in whom bleeding had not been practised until after that period. But, as there must be either some positive mistake here, or there were certain influencing circumstances in the one set of cases which did not operate in the other set, we may pass this part of our subject over; and shall now advert, for a moment, to the view which the tables of M. Louis present to us, of the relative duration of the disease in different sets of cases, according to the treatment which had been employed. It may be useful to premise, that some physicians are too apt to be guided in their practice by the alleged superior dispatch of one mode of treatment over another, forgetting the old adage, "sat cito, sat bene." Let it be remembered that, in the treatment of a disease like pneumonia, it matters but little whether the patient be "on the doctor's list" one or two days, more less. Our object is to get him well; speedily well, if possible, but at all events well. In our attempts to make a speedy cure of a disease, how often are we not only frustrated, but even punished with an unusual delay. Some years ago, a proposal was made to treat gonorrhœa, from the commencement, with injecting a strong solution of nitrate of silver into the urethra; but it was soon found that, except in a few cases, in which the discharge was certainly very speedily arrested, the medium duration of the caustic treatment was considerably greater than when merely demulcent drinks and rest had been employed. Equally, and much more seriously true, is the remark, in respect to the treatment of typhus fever. Indeed we should be almost inclined to say, that all attempts at a very speedy, or "coup-de-main" sort of treatment ought to be deprecated, in fevers of every kind. It would seem that they have an appointed course to run, and that any effort to arrest this suddenly only accelerates the mischief, or renders it more deeply and more immoveably seated, and, therefore, more difficult of removal. Let it not be supposed that we are unfriendly to bold and decisive therapeutic measures—far from it: all that we contend for is, that it is of infinitely greater importance, and it is infinitely more creditable to a physician, to save 20 patients in any manner, than only 19, although the cure, in each of these 19 cases was surprisingly rapid. The great art of an accomplished physician is, on the whole, rather to *lead* a disease through its stages to a favorable issue, than to attempt to strangle it at once. The latter attempt may indeed sometimes succeed to admiration; but, if it does not, the very attempt must inevitably entail mischief on the patient.

From these general and discursive remarks, let us now attend to some of the details of M. Louis' memoir, respecting the treatment of pneumonia by large doses of the tartrate of antimony. We are informed that in sixteen cases, so treated, the duration of the disease, was longer by three days than in those, where the medicine had not been used; but that the mortality did not exceed the proportion of one in six patients. The latter part of this statement is much more to the purpose than the former part. We should require to know the exact nature, and the special particularities of each case, before we could safely draw any satisfactory conclusion that the duration of pneumonia, when treated with the tartrate, is usually longer, than when it had not been used. But if we object to M. Louis' opinion on this score, we have much stronger objections to the following doctrine, respecting the therapeutic effects of blood-letting: viz. " que la saignée n'a que peu d'influence sur la marche de cette maladie, et que son influence n'est pas plus marquée dans ceux où elle est copieuse, et repetée, que dans ceux où elle est unique."

Now the very data of M. Louis' own memoir do not warrant the conclusion, that venæsection, practised to a large amount and frequently repeated, has but little influence on the progress of pneumonia; for in the majority of his cases, neither was the quantity of blood drawn very large, nor was the operation frequently repeated. All that can be fairly deduced from M. Louis' researches, is that small bleedings have not had " des effets très-marqués sur la marche et les symptomes de cette maladie." As to the effects of very copious bleedings, especially of such bleedings frequently repeated during the first days of a pneumonic attack, the practice of M. Louis does not entitle him to judge. The clinique of Professor Bouillaud, however, can bear undisputed evidence to the efficacy of such vigorous treatment.

M. Donné refers to some reports of cases which he saw treated in the Hôpital de la Charité during the years 1832 and 3, on which he founds this judgement. Out of 23 cases, only one proved fatal. He adds " je dois avouer que l'energie avec laquelle M. Bouillaud a traité ces maladies, m'a plus d'une fois ebranlé, et il n'a fallu rien moins que le succes eclatant qu'il a obtenu pour me rassurer, et me convaincre." The extent of the venæsections, and the shortness of the intervals between each operation, were assuredly the cause of this great success. When the case was at all severe, M. Bouillaud's usual practice is to bleed to the amount of 20, or 24 ozs. say, in the morning; to repeat the operation in the evening, and during the interval to order 15, or 20 leeches to be applied over the seat of the pain; the bleeding from the leech-bites to be generally encouraged by the application of cupping-glasses over them. On the following morning another similar bleeding from the arm was usually practised; again repeated in the evening, and the leeches were re-applied. On the third, fourth and fifth days, one venæsection was very generally practised. On the whole, the average number of bleedings was from 4 to 7, each being copious; and the number of leeches applied from 60 to 80; and all this, in the course of the first 5 or 6 days.—*Archives Générales.*

---

### Interesting Case of Chronic Pleurisy, accompanied with Fistulous Openings in the Left Lung, Pneumothorax, and Loose Osseous Connexions within the Pleura.

Although chronic pleurisy is most generally the consequence of the acute form of the disease, yet sometimes it is primary, and then its approach is often so obscurely marked, that it may have existed for a length of time, and extensive effusion may have taken place into the pleural cavity, before the patient is at all aware of any mischief. His general health may not have suffered so much, as to prevent him from continuing his ordinary avocations; the pain may be very trifling, or it may be altogether absent; the cough is usually irregular and never very troublesome at first, and the breathing is often free from any marked inconvenience. Chronic pleurisy affects sometimes the whole of one side, at other times it is limited to a small extent. Very rarely both pleuræ are affected at the same time. The symptoms are much influenced by the extent of the disease. When it has existed for some time, and a large extent of pleural surface has been affected, the auscultatory signs are usually very expressive, and easily recognised. The sound on percussion over the whole, or the greater part of one side is dull, and the respiratory murmur and resonance of the voice are very feeble and indistinct. The patient not unfrequently complains of a sense of weight on the affected side; and when the effusion is large, or has been of long continuance, this side may be obviously fuller and more expanded than the other one. In some cases the intercostal spaces are considerably more separated from each other than in a state of health, and not unfrequently they are very manifestly more projecting. Some physicians have asserted that they could discover the presence of fluid by tapping the chest with the fingers, as we do the belly in ascites; but this must be very rarely the case, as Chomel states that he has often tried the plan, but has never succeeded in the attempt. The state of the respiration should be always diligently examined. When the patient is perfectly quiet, there is often no disturbance of this function; but any exertion, as quick walking, loud talking, coughing, &c. brings on a sense of distressing tightness and oppression, from which he does not recover for some time. Firm pressure on the abdomen, or on the affected side of the chest will produce the same result. The cough is generally more or less irregular, both in its frequency and in its severity; the expectoration is in most cases tenacious and of a mucous character. The position of the patient in bed ought never to be neglected:—he generally lies on the affected side, on the back, or in an intermediate posture.

The most obvious constitutional symptoms are general pallor of the surface, a certain puffy, or bloated appearance of the features, a sense of coldness and numbness of the extremities, an increasing diminution of the muscular and mental energies, and a looseness and flaccidity of the fleshy parts.

In primary chronic pleuritis, there is, as we have already mentioned, often an entire absence of any febrile movement; but in that variety of the disease, which succeeds to an attack of the acute form, there is generally a tendency to evening fever, followed by partial sweats during the night. The severity of the feverish attacks is very probably influenced, at least to a certain degree, by the nature of the effused fluid. When this is entirely of a serous character, as is usually the case in primary chronic pleuritis, there is little tendency to fever; but when it is puriform, there is always more or less of an irregular hectic pyrexia.

The duration of chronic pleurisy varies from two months to one or two years. During its continuance there are very usually occasional remissions and exacerbations of the symptoms, before the final tendency, whether favourable or not, is fairly established. It is rather by the state of the general health, than by the presence of any local symptoms, that our prognosis ought to be guided. When the decay of the strength is gradual and progressive, when the breathing becomes more and more distressed, when the pulse becomes more and more quickened and irregular, and especially if any tendency to anasarca, or to diarrhœa supervenes at the same time, our hopes of an ultimate cure can be very feeble indeed. On the other hand, when the general health improves, and the cough and dyspnœa become less and less troublesome, we may confidently expect that the effused fluid will become gradually absorbed. The progress of the absorption may often be ascertained by the gradual diminution of the dimensions of the affected side, which then becomes flatter and flatter, and even sometimes is visibly drawn in. These changes are most conspicuous in old cases, where the effusion has been large, and the compression of the lung has been so long continued that it remains contracted and shrivelled. Absorption is not however the only method, which Nature employs to remove the effused fluid. In some cases, especially in those, where the fluid is of a purulent character, the fluid may work itself an exit, either through the pulmonary pleura, and the substance of the lungs into some of the bronchi, from which it is rejected by expectoration, or through the thoracic parietes outwardly. In the first of these cases, a patient affected with chronic pleuritis, who may have been suffering for several weeks, or months with a troublesome dry cough and scanty expectoration may all on a sudden be seized with a profuse expectoration, or we may rather say, a vomiting of purulent matter, which continues more or less abundantly from the date of the seizure.

The phenomena of the second case cannot be mistaken. One or more fistulous openings at some part of the thorax make their appearance, and from these a purulent, or semi-purulent discharge exudes, especially during any effort of expiration, as for example, immediately after coughing, sneezing, or the like. The introduction of a probe will render the diagnosis more sure: if it can be passed to a considerable depth, and its point be then freely moveable, it is reasonable to believe that it has entered the pleural cavity. Chomel has witnessed one or two cases in which both of these lesions took place nearly at the same time; so that the escape of the effused fluid, partly by expectoration and partly by external draining, occurred almost simultaneously. M. Lerminier in his Clinique, has reported an interesting example of this double lesion.

Of the two lesions, the internal one, or that of ulceration of the pulmonary pleura and parenchyma, is of more rare occurrence, than the external one. Were we to trust merely to the reports of patients we might judge otherwise; for it is by no means unfrequent to hear patients talk of their having vomited large quantities of pure pus, on the decline of a fluxion of the chest. The physician must be on his guard not to be misled by such statements. Unless the supervention of a profuse expectoration of pus has been quite sudden, and as it were instantaneous, the mere circumstance of the sputa in any case being puriform and in large quantities, is not sufficient to warrant the suspicion of a pulmonary fistula having taken place. Chronic bronchitis, with dilatation of the air-tubes, is often accompanied with a copious puriform expectoration. The prognosis in either case must be on the whole very unfavorable. Recovery is not impossible; but it is certainly very uncommon.

In some cases, the patient has been immediately suffocated by the rapid escape of the fluid into the bronchi. The chances of recovery are certainly much diminished, when the quantity of the discharge, whether it finds an outlet internally by the bronchi, or externally through the integuments, is very profuse. We have not yet alluded to the auscultatory signs of the lesions which we have been considering.

Our readers will find some very useful remarks on this branch of symptomatology in our Review of Louis on Phthisis in the present Number. The sudden occurrence of percussion eliciting an unusually resonant sound in a part, where previously the sound was quite dull, while the respiratory murmur continues faint, or is altogether inaudible, the amphoric blowing, and the metallic tinkling sound, are, when present, very satisfactory evidences of the nature of the lesion which has taken place. It is however necessary to know, that in some cases of perforation of the pleura, especially where the chronic pleuritis has been very protracted, and where the adhesions are very extensive, the escape of air into the pleural cavity may be rendered impossible, and the auscultatory symptoms must necessarily be absent, or at least very indistinct. The prognosis in chronic pleuritis, is on the whole decidedly unfavorable. Of eleven cases treated by M. Chomel in the wards of La Charité, five proved fatal. Even when the absorption of the fluid is complete, the patient seldom recovers his health and strength entirely. He remains subject to dyspnœa, occasional cough, &c. The prognosis is more unfavorable in consecutive, than in primary and idiopathic chronic pleurisy; the effused fluid in the former case being usually of a more puriform character, and the false membranes being thicker, and frequently also more extensively deposited.

The morbid changes which these false membranes may undergo, are among the most interesting themes of enquiry to the scientific pathologist. Soon after their deposition, they become provided with blood-vessels, acquire a particular organization, and may become subject to diseases, like other organized structures; for example, to the effusion of a serosity, between their layers; to the deposition of new lymph upon their surfaces; to hæmorrhages, as appears from clots of blood having been found in their substance; to gangrene, according to M. Laennec; to tuberculous deposition, not a very uncommon phenomenon; and lastly to the production of fibro-cartilaginous, and osseous concretions.

It is unnecessary to enlarge upon the treatment of chronic pleurisy, as no good physician, after once ascertaining its existence, can hesitate about the remedies he ought to employ. When the effusion has taken place, the frequent application of blisters over the affected side, and the internal use of mild mercurials, with laxatives and diuretics, are the means which have been found most generally beneficial. But we again repeat that to form a correct diagnosis is infinitely more difficult, and infinitely more important, than the mere knowledge of the most approved remedies. Occasional pain, cough, dyspnœa increased on any bodily exertion, sense of weight and oppression on one side, inability to lie comfortably on the sound side, frequent pulse, rapid breathing; these symptoms, when associated with dullness of sound on percussion, feebleness or total absence of the respiratory murmur and of the resonance of voice, or in other cases the œgophonic sound, must suggest the existence of pleuritic effusion. When perforation of the substance of the lung is co-existent with effusion, the peculiar "tintement metallique," and the amphoric blowing usually become perceptible. We shall now detail two interesting cases, which illustrate many of the preceding remarks.

CASE 1.—*Chronic Pleurisy, with Pneumo-thorax, from a fistulous opening in the lung.* A man 66 years of age, was received into the Bicetre on the 12th of August, 1834. Four years previously he had sustained a violent blow on the left and middle parts of the chest. An attack of pneumonia was the consequence; and according to the patient's report, he some time afterwards expectorated a large quantity of pus. From that period, the left side has sensibly contracted, and the spinal column, towards the upper dorsal part, has been gradually inclining to the right side. When he was first admitted into the Infirmary, his chief complaints were extreme weakness, and pains in the loins and lower extremities. But as the expectoration was truly purulent, and as there was regularly an evening febrile attack, and also profuse perspiration during the night, it was suspected that serious inward mischief was existing somewhere.

The chest was therefore attentively examined with the stethoscope; and unequivocal signs of the presence of vomicæ in the summit of the left lung were readily discovered. Pectoriloquy and cavernous respiration were very audible. Percussion over the same part elicited a more sonorous sound than is heard in health. The posterior and inferior parts of the chest emitted a dull sound on percussion, and there no respiratory murmur could be heard. The absence of œgophony, and more especially the persistance of the dull sound on percussion, and of the defective respiratory murmur, over a space of uniformly the same limited extent, argued against the supposition of there being any effusion into the pleural cavity. The patient died during the third week after his admission into the hospital. The symptoms had not undergone any decided change, or very marked aggravation, but the evening fever and the night-sweats rapidly exhausted all the powers of life.

*Dissection.* The right side of the thorax was observed to be much more expanded than the left side; the ribs were unusually apart from each other. The left side was considerably contracted; the fifth, sixth, and seventh ribs of this side were almost in contact with each other. On examining the lungs, the upper lobe of the right one was found to contain numerous crude tubercles. In the upper lobe of the left lung, the tubercles were more numerous, and there were also two small vomicæ, which were empty, and lined with a mucous membrane. These vomicæ were separated from the pleura by a layer of sound pulmonary substance, a circumstance which must have prevented any communication between the excavations and the pleural cavity. The lower two-thirds of the left lung were pushed up and compressed by a large quantity of puriform fluid, contained within the pleural bag. The external or costal wall was lined with a false membrane, several lines in thickness, of a yellowish colour, and of a firm, semi-cartilaginous consistence. Two inches below the summit of the left lung, there were discovered five small apertures, penetrating through the pulmonary pleura at this part, and leading directly into some of the bronchial divisions, but not into either of the vomicæ. These apertures were large enough to admit readily a crow-quill; and the length of the passages leading from thence into the adjoining bronchi was from a third to half an inch. The purulent matter contained in the pleural bag was "lié, consistant, homogene, peu odorant, d'une couleur blanche grisatre." On comparing it with the expectorated sputa, the two were found to be quite alike. Lying quite loose in this purulent fluid, were found upwards of a dozen semi-osseous concretions, of various dimensions and forms. The largest were of the size of a 40-sous piece; they were uneven and rough on their surfaces and on their edges; and here and there they exhibited an appearance, as if they had been sprinkled with a cretaceous powder. In a few of them were observed small openings, very like the orifices for the passage of the nutritious vessels of the bones. Nothing unusual was found in any of the other viscera.

*Remarks.* It becomes a question of interesting consideration, how long the ulcerated openings in the left lung, and the consequent communication between the pleural cavity and the vomicæ had existed. Had they been present ever since the purulent expectoration had commenced? The case, in some respects, resembles some which have been published by Bayle, in his treatise on phthisis, in which he points out the very great resemblance between some examples of chronic pleuritis, and of genuine pulmonary consumption. The diagnostic mistake which was made in the preceding case was certainly very excusable, seeing that there was a co-existent tubercular disease of the lungs. The absence of œgophonic sound, and of any sign which might indicate that the level of the contained fluid changed with the change of position, was attributable to the circumstance of the effusion being confined, and, as it were, encysted, within narrow limits, by the adjoining adhesions of the opposing pleuræ. This case proves also that the metallic tinkling sound is not an uniform symptom of a fistulous opening from the lungs into the cavity of the pleura, complicated with pleural effusion. Whether this was owing to the circumstance of no fluid dripping from the apertures, into the effused fluid in the pleural bag, or of the effused fluid reaching nearly to the level of the apertures—or, lastly, of the apertures being (according to the explanation of the metallic tinkling recently proposed by M. Beau) not beneath the surface of the contained fluid, we are not prepared to say.

The theory of M. Beau is highly ingenious:—He supposes that the "tintement metallique," or, as he prefers calling it, the "tintement bullaire," is attributable to the escape and passage of air-bubbles from the fistulous opening, through the effused fluid, these bubbles bursting when they reach the surface of the fluid. The bursting of the air-bubbles being, according to him, the cause of this peculiar sound, it is obvious that, if Beau's explanation be correct, this very peculiar sound cannot be present, unless the ulcerated opening of the lung be situated under the level of the effused fluid.

M. Bricheteau, in whose service, at the Hospital of Necker, M. Beau acted as clinical clerk, seems inclined to adopt his pupil's views. In his late work, "Clinique Medicale," he states that he has frequently repeated M. Beau's experiment (this consisted in blowing gently through a small tube, introduced into a flask half filled with a fluid; the air-bubbles, as they rose to the surface and burst, "simulait, à l'oreille de l'observateur attentif, le tintement metallique") and testifies that "toujours le bruit, obtenu par l'explosion de la bulle d'air, nous a paru conforme à celui du tintement metallique."

It is worthy of notice, that the late M. Dance had anticipated M. Beau, in the explanation given of this curious auscultatory sign—*vide* the late edition of the Dictionnaire de Médecine.

The circumstance of these two observing physicians having, quite unknown to each other, arrived at the same conclusion, is well calculated to confirm its probable accuracy.

The case now recorded confirms the accuracy of the remark, that pneumothorax is of much more frequent occurrence on the left, than on the right side of the thorax. The cases of M. Louis (reported in the review of his work on phthisis, in the present Number) lead to the same result; and from a table of forty-nine cases, the histories of which had been collected by Dr. Renaud in 1830, it appears that, in not fewer than thirty-two of them, the disease existed on the left side.

With regard to the loose and undetached osseous concretions found in the purulent effusion, it is difficult to propose any satisfactory explanation of their origin. It is well known to every pathologist, that partial ossification of portions of the pleura is not of very rare occurrence; but few, if any cases of osseous concretions lying loose within the pleura, and unconnected with this membrane, are on record: occasionally they have been discovered in other serous cavities. In the art. "Cartilage accidentale," of the Dictionnaire des Sciences Médicales, an article which was written by M. Laennec, it is stated that some anatomists have met with osseous concretions "dans les cavités memes de l'arachnoide (the cerebral ventricles?) where they floated quite loose and unadherent.

Cartilaginous, and even osseous concretions have been not unfrequently found in the sac of the tunica vaginalis testis. The chemical examination of the concretions which M. Prus found within the pleural cavity, was performed by M. Guibourt, an expert analyst. The following is the report of his examination.

The substance of the concretions was of a yellowish-white colour slightly translucent, irregular, tuberculated, presenting, in its interior, numerous small cavities, and consisting of an apparently fibrous structure. When pulverized, it emitted an extremely offensive odour, like that of intestinal fat. On exposure to heat, it became black, and burned imperfectly, without producing any flame, or becoming at all melted; the smell was similar to that emitted from mucus, when heated. Æther dissolved it very partially; and, when the solution was evaporated, a fatty, and somewhat saponaceous residue was deposited. Cold water had very little effect upon the substance; a small quantity of saline matter only, chiefly muriate and sulphate of lime, being taken up. Boiling water extracted a small portion of a mucus-like matter. The quantity removed by these agents, used successively one after the other, was very minute; 2·21 grammes (each gramme consisted of nearly 15½ grains) was only reduced to 2·065. This residue, being then exposed to a white heat, in a platinum crucible, it lost exactly one-fourth of its weight, and there remained a white product, which effervesced feebly

P 2

on the addition of muriatic acid. The result of the analysis may be stated, as follows :—

| | | | |
|---|---|---|---|
| Phosphate of lime, | 1·085 | = | 49·1 |
| Carbonate of lime, | 0·465 | = | 21·1 |
| Insoluble mucus.. | 0·615 | = | 27·8 |
| Fatty matter .... | 0·040 | = | 1·8 |
| Soluble salts .... | 0·005 | = | 0·2 |
| | 2·210 | | 100·0 |

Berzelius' analysis of human bone is subjoined, in order that we may be able to form a comparison between it and the concretions found within the pleural sac.

| | |
|---|---|
| Gelatine .......... | 32·17 |
| Vascular tissue ..... | 1·13 |
| Phosphate of lime... | 53·04 |
| Carbonate of lime... | 11·30 |
| Carbonate of magnesia | 1·16 |
| Muriate of soda .... | 1·20 |
| | 100·00 |

The chief difference in the composition of the concretions from that of bone, appears to consist in the substitution of the insoluble mucus in the place of the gelatine, and in the double proportion of the carbonate of lime.

The composition of the pleural concretion agrees very nearly with that of many accidental ossifications; for these latter are almost always found to contain a quantity of insoluble mucus, and little or no true gelatine.—*Révue Médicale.*

CASE 2.—*Chronic Pleurisy—Empyema bursting outwardly, through the Diaphragm and the Abdominal Parietes.*

Nicolas Constanza, 40 years of age, had, for the preceding 20 years, suffered repeated attacks of ague, which had left behind an enlarged and indurated state of the spleen. Fifteen months before his death, he began to experience sharp lancinating pains in the lumbar region of either side, which baffled all attempts at relief. In April, 1834, the pain in the region of the spleen increased in severity, and a considerable fulness was observed in this part. The symptoms of hectic fever coming on, he was admitted into the Hospital of Incurables, at Florence. As the swelling was supposed to depend upon a deep-seated suppuration, poultices were applied for some weeks. At length it pointed so distinctly, that the surgeon was induced to open it with the lancet. A large quantity of dark-coloured, sanious pus was discharged from the wound; but no sooner had most of the pus been evacuated, than the patient suddenly lost his voice, and his breathing became greatly distressed. The hectic symptoms rapidly increased in severity after the operation; the abdomen became swollen, and excessively painful, especially towards the right iliac region, the urine was hot and burning, the respiration hurried, the tongue red and parched, and, in short, all the symptoms of an acute peritonitis rapidly developed themselves, and death speedily followed.

*Dissection.* The spleen was found to be perfectly normal in structure; but it was situated rather lower in the abdomen than usual. The abscess, which had been opened, was found to communicate, by a perforation through the diaphragm, with the left pleural sac, where a vast collection of purulent matter, similar to what had escaped outwardly by the wound, existed. The left lung was extremely shrivelled, and forced up against the posterior mediastinum. In the purulent matter, numerous coagula of blood, such as we find in aneurismatic sacs, were floating about. The costal pleura was greatly thickened and indurated, and this, as well as the pulmonary pleura, was of a dark colour. The diaphragmatic pleura also was much thickened, and was of the same colour. The right lung, the heart, and its large bloodvessels were normal. The state of the abdominal viscera is not described.

The preceding case is curious, as shewing that an abscess, formed in the left side of the chest, may occasion but little pain or swelling in the part immediately affected; and that the chief seat of the local symptoms may be in some portion of the abdominal parietes.

It is interesting, also, as a proof of the beautiful provision made by Nature against internal effusion; for while the ulcerative absorption of the diaphragm was going on, this muscle had contracted an adhesion to the walls of the abdomen, and had thus prevented the escape of the purulent contents of the pleural cavity into the abdomen, which otherwise must have been inevitable.—*Filiatre Sebezio,* 1834.

---

### Cure (?) of a Vomica, or Abscess in the Lungs, by Bleeding.

The following case is reported in the Journal Hebdomadaire, as having occurred recently in the wards of La Charité, under the care of M. Bouillaud, who is generally acknowledged to be a most accomplished stethoscopist.

A young man, 22 years of age, was admitted into the hospital, on the 17th of last September. He had been ill for upwards of two months; his illness commencing with the symptoms of a common cold, and characterized in its progress by a harassing cough, which was at first dry, and then, accompanied with expectoration of a fetid muco-purulent matter occasionally streaked with blood, by general weakness and emaciation, and by most of the symptoms of an incipient hectic.

On the 30th of July he entered into the Hôtel Dieu, in consequence of a troublesome diarrhœa He remained in that hospital for six weeks, during which time the pectoral symptoms had not become at all mitigated. When admitted into La Charité he was pale and languid; the cough was frequent; the sputa filthy, of an offensive smell, and amounting in quantity to twice the contents of a "crachoir" in the course of 24 hours. The summit of the right side of the chest generally, sounded dull on percussion. On examining this region with the ear, a cavernous blowing, alternating with a gurgling sound, might be distinctly heard; and when percussion was employed directly over the part where these sounds were heard, a bruit like that caused by striking a cracked vessel was elicited. On the left side of the chest, the respiratory murmur was puerile, and the resonance from percussion normal. M. Bouillaud ordered the patient to be bled to sixteen ounces, to lose the same quantity of blood by cupping between the shoulders, and to live on a mild diet of broth and vegetables. Considerable relief to the cough, and diminution of the quantity of the sputa followed the bleedings: the engorgement however of the summit of the right lung still continued. After the lapse of a fortnight, another venæsection to the same amount was practised. On the day following, the dull sound on percussion of the summit of the affected lung was found to be greatly diminished; and it was with the view of dissipating the engorgement altogether, that, eight days afterwards, the cupping-glasses were ordered to be applied immediately over the diseased part. From the date of this last bleeding, the patient experienced the most decided amendment; for, in the course of two weeks, the cough and expectoration had entirely ceased. The sound now elicited by percussion at the summit of the right lung, was clear and resonant. The cracked-vessel sound was still perceptible lower down on the same side, but it was less extended than it was at the commencement of the treatment. The patient speedily began to recover his strength and flesh, and he gradually advanced to a complete recovery.*

*Remarks.* "It is not after the manner," says the French reporter, "of the ancients, who trusted to the rational symptoms only in their diagnosis of diseases, that we announce the above successful treatment of a case of genuine pulmonary phthisis. We recognized its existence by indisputable physical signs; signs which are at once positive, and the knowledge of which is not the privilege of any one physician, or sect of physicians in particular, but is easily and satisfactorily within the reach of

---

* The bruit of the "pot-feléc," a sign of an old cavernous excavation, remains, we are told, to the present day, although all the other symptoms of such a lesion have disappeared.

all the world! Moreover it was not one person only who proved the true nature of the disease in our patient: it (the disease) was 'reconnue, mesurée, calculée, et notée,' as very curious by a numerous auditory of pupils and physicians, foreign as well as domestic. The case, however remarkable, is not cited as rare or very singular; but with the hope of convincing the profession of the unequivocally curative effects of the treatment which was adopted. The general amendment which followed each bleeding was too obvious to escape the notice of any one present, and in short 'emission sanguine est la seule medication qui peut etre dirigée directement contre la nature de cette affection.' What! will any one in the present day maintain that the act of tuberculisation is not analogous to that of inflammation? Let them scrupulously, and with minds free from prejudice, enquire of patients who are labouring under tubercular disease of the lungs, and they will find, that almost every one of them has, at some period or another, had fluxions or neglected colds, and has been more or less exposed to the influence of atmospheric changes. If it is alleged, as an argument against the inflammatory origin of pulmonary tubercles, that they are frequently coexistent in several tissues, or organs of the body, may we not reply, that this feature of dissemination is true of other diseases, which are undeniably genuine phlegmasias? Is not pleurisy often coexistent with pericarditis, peritonitis, or with inflammatory rheumatism? Does not erysipelas very often develop itself in several parts of the body successively? The mere circumstance, therefore, of more than one or two organs being simultaneously affected with tubercles, (and be it remembered, this complication is very far from being of constant occurrence,) cannot therefore be fairly adduced to disprove the phlogistic origin of tubercles. It is usually in old protracted cases that we find several organs similarly affected at the same time; and all physicians are aware that the duration of diseases has a great influence on their type and character (forme.) When an acute cerebritis terminates in the formation of an abscess, small although this may be, how complex is the series of symptoms which precede death; whereas, when such an abscess is formed very slowly, we know that life may be protracted for years. Let us therefore never forget that it is irrational to call every thing, which we cannot well explain, impossible. Plura enim in morbis fiunt, quæ observare tantum licet, minime vero intelligi."—*Lancette Française.*

We have described the details of the preceding report at much greater length than it merits, either from the practical importance of the case which is described, or from the reflections to which it has given rise in the mind of the narrator. Our chief motive has been to exhibit to such of our readers as are not in the habit of perusing the continental medical literature, a specimen, by no means of unusual occurrence, of the spirit which too often pervades the reports and the reasonings of many of the French physicians.

The case is announced to be one of cure of genuine tubercular phthisis, in the last stage of the disease, when the tubercles have softened, and vomicæ have been formed in consequence. Now we (*Rev.*) have strong doubts whether the diagnosis was correct. Certainly the details of the report do not warrant the cautious critic to pronounce the case to be one of genuine phthisis; and it must be from these details only, that we or any other readers can deduce our opinions.

There is no mention made of the constitution or habit of body of the patient: we are not told whether the phthisical tendency was hereditary or accidental; many of the most pathognomonic "rational" symptoms of confirmed pulmonary consumption seem to have been altogether absent; and even some of the signs deduced from auscultatory examination, bronchophony, and pectoriloquy for example, are not even alluded to in the report. The signs, the existence of which is adduced as sufficient evidence of the malady, are the "cavernous blowing sound, alternating with a gurgling râle heard with the stethoscope, and the dull resonance elicited by percussion over the summit of the right lung, and the cracked-vessel sound lower down, on the same side of the chest." Now the cavernous and gurgling bruits, although justly of very suspicious import, are by no means of themselves, decisive indications of a purulent excavation. When any of the bronchial tubes become much dilated, and especially when at the same time they lie near to the surface of the chest, the respiratory noise may assume a well marked cavernous character; and the cough and the ordinary mucous rattle may present the bronchial or cavernous character. Stethoscopists are quite aware how extremely difficult it is to draw the distinction between a loud mucous rattle, and the "gargouillement," which has been improperly supposed by some to be inevitably characteristic of the existence of an ulcerous excavation.

With respect to the signs obtained by percussion, we need only state, that the dull resonance indicates indeed an obstructed and hardened state of the subjacent lung, whether this state be produced by the deposition of tubercles, or from any other cause; and that the other sound, the cracked-vessel sound, is acknowledged by Laennec himself to be a very ambiguous sign, and on the whole, one of little value. Such are some of the reasons, which make us hesitate to receive the preceding case, as an example of a genuine phthisical vomica successfully treated. We have not alluded to the treatment, to which the credit of the cure is ascribed. A patient pronounced to have a suppurating cavity in the right lung, and who was pale, weak and emaciated, and without a single symptom (at least none is mentioned) of an inflammatory affection, is bled and cupped twice, losing in all 64 ounces of blood, and in the course of a few weeks is declared to be cured! Need we make any comments? We lately read the reports of some cases adduced by Sir C. Scudamore, as examples of confirmed phthisis cured by iodine, &c. inhalations! The *successful* practice of the English, as well as of the French physician, is, we much fear, limited to their own patients. Their professional brethren somehow or other can never imitate them.

---

### Treatment of Pertussis by Revulsives.

It was Autenrieth, who first distinctly pointed out the great advantages which may be obtained in the treatment of numerous cases of hooping-cough, from the use of epispastics. Of these he gave the decided preference to the strong tartar-emetic ointment rubbed on the epigastric region, until a very considerable irritation, and even a painful ulceration are fairly established. M. Corsin employed this practice extensively in an epidemic of the disease, which prevailed in Petersburg several years ago, and he derived, he tells us, most decided benefit from its adoption. He was led however to modify the formula for this ointment recommended by Autenrieth, and to combine some anodyne with the tartar-emetic. Subsequently he has preferred the use of a plaster, which he has found still more effectual than any other application. This plaster is composed of two parts of plaster of hemlock, one of Burgundy pitch plaster, and one of diachylon, to be spread on leather, the surface of the plaster to be then sprinkled with 6, 8, 10, or 12 grains of the antimonial tartrate. It may be applied either to the epigastrium, or between the shoulders. Usually in the course of one, or two days, it induces considerable irritation; the skin is first reddened, and then a free eruption of pustules is brought out. The patient must continue to wear the plaster, until either the cutaneous irritation is so troublesome, that he cannot bear it any longer, or until a decided relief to the hooping-cough is obtained.

*Case* 1. A boy, 8 years of age, of a scrofulous constitution was suffering from a severe attack of inflammatory pertussis, when Dr. C. was called to his assistance. A vigorous antiphlogistic treatment was immediately adopted; and when the active symptoms were once subdued, a plaster made of the ingredients mentioned above, (2 drachms of empl. conii, and one drachm of Burgundy pitch, and the same quantity of diachylon), and sprinkled with eight grains of tartar-emetic was applied between the shoulders. In 24 hours, it had occasioned a most troublesome itching of the part, which was inflamed, and covered with numerous vesicles; in other 12 hours, it required to be taken off, and the surface was dressed with simple cerate. The distress of the breathing was greatly relieved; the paroxysms of cough were already less frequent and severe, and the child was in every respect much better. On the following fifth day, the plaster was reapplied to the same part: the cutaneous irritation induced was speedily even more considerable than it had been before. Fortunately the relief afforded was proportionally great; for, from this date, a most decided amendment was conspicuous, and in the course of a few weeks, the child was free of every symptom of his troublesome disease. It deserves to be noticed that the internal use of the belladonna had been suspended after the application of the plaster, and that the only medicines exhibited were mild demulcents.

*Case* 2. The age of this patient was four years. During the preceding Summer she had been affected with an obstinate porriginous eruption on the scalp; and, as this had disappeared, hooping-cough set in and had affected her general health greatly, not only by the frequently repeated and severe paroxysms of coughing, but also by an almost constant irritability of the stomach, so that all food was rejected, as soon as swallowed.

A plaster of hemlock and Burgundy pitch, sprinkled with five grains of the antimonial tartrate, was applied between the shoulders, and a mixture composed of a mild bitter infusion, with the addition of syrup of poppies and of cinchona powder, was ordered to be given in repeated doses during the day. In 24 hours, the plaster was removed, for it had already induced a copious eruption of pustules. The sickness had quite ceased; and the cough was greatly mitigated. No second application was necessary; and this girl in the course of a week, or two, was pronounced to be well.

Several other cases, all of which evince most satisfactorily the admirably curative effects of the plaster, we have described, are detailed by Dr. Corsin. He is inclined to explain the modus operandi of this external treatment, on the principle, that there is in most cases of hooping-cough, a tendency to the occurrence of some cutaneous eruption, whether this tendency be of spontaneous development, or whether it is the result of the retrocession of a pre-existent exanthem. It has indeed been often remarked that the severity of pertussis is frequently much mitigated on the supervention of impetigo, or porrigo.—*Lancette Française.*

---

### M. Andral on Gastro-Enteritis and Follicular-Enteritis.

By far the most strongly marked cases of this phlegmasia, in its active or acute form, are those which are induced by poisonous doses of acrid and corrosive substances. Almost immediately after they have been received into the stomach, the patient experiences a burning pain in the epigastrium and at different points of the abdomen; he is tortured with an insufferable thirst; the belly speedily becomes distended, perhaps irregularly so, and hence it has often the appearance of being retracted in some places, and inflated at others; retching and vomitings ensue, and then follow purgings, at first of a bilious or serous discharge, and subsequently of a bloody fluid, or even of pure blood. The tongue becomes dry, smooth, red, and chapped on the surface; and in some cases a sanguineous fluid oozes from the fissures. The pulse is usually strong and full, and the skin is hot at first; but the one soon becomes feeble and thready, and the other cold and clammy. After a certain time, the symptoms of local suffering gradually abate, and may then cease altogether, so that a physician being called in at this stage of the case, may be very easily deceived as to its true nature, and be led to suppose that it is one of simple typhoid fever: the constitutional depression, the weak faltering pulse, the hurried breathing, the furred tongue, the tendency to delirium, or coma;—are not these the veriest outward signs of typhus? They are so; but not exclusively or essentially of idiopathic typhus; but rather of a certain stage of gastro-enteritis, whatever be the cause, which may have given rise to this morbid state. Tartra mentions in his thesis several cases of poisoning from nitric acid, in which the gastro-enteric distress was indicated, not by any local symptoms, but only by the presence of adynamic fever. When gastro-enteritis is of spontaneous origin, it is very generally preceded by certain derangements of the system, and more especially of the digestive functions: the appetite is usually much impaired; sometimes, but this is rare, it is more voracious. A capricious state of the appetite is perhaps of the most frequent occurrence; the patient feeling at times the most intolerable hunger, and at other times, an utter want of desire for food. The bowels are very generally either constipated, or they have a tendency to be purged. Headache, universal lassitude, and pains in the limbs also are common precursors of gastro-enteritis; and indeed in no disease, is the truth of Hippocrates' maxim more frequently realised; "lassitudines sponte abortæ morbos denuntiant." The seat of pain in this disease varies, as a matter of course, with the exact seat of the inflammation; but it is worthy of notice, that in some cases it is fixed to a point behind the ensiform cartilage, extending perhaps from this point along the tract of the œsophagus to the back of the throat, so that the physician may very naturally suppose that the cardiac end of the stomach was chiefly affected; and yet on dissection no traces of any phlegmasia may be discovered at this part. The peculiar appearances of the tongue in this disease, ought to be well understood by medical men: we shall therefore describe them at some length, premising that there is usually experienced a sense of distressing heat in the mouth, that the lips are parched, red, chapped, and bleeding, and that the gums are sometimes invested with a thin layer of coagulable lymph.

The sensibility of the tongue is frequently increased, and the patient complains of a burning, or pricking sensation at the point; sometimes it is so distressing, that the mere contact of any substance, however emollient, gives pain. The apex may be drawn out and thus present a more lanceolated shape than usual; and the whole organ may be thickened, so that its movements are more difficult than in health. The colour of the surface of the tongue is variable; sometimes it is not at all changed, whatever be the seat of the phlegmasia; but this negative sign is applicable chiefly to those cases, in which the lower part only of the intestinal canal is affected. In the greater number of instances the colour of the tongue undergoes certain modifications, which are not indeed always commensurate with the violence of the inflammatory action, but which are greatly influenced by the state of the innervation of the whole system. Sometimes it is intensely red, and this redness may be either over the whole extent, or only in patches, or insulated points: at other times, it has rather a brown colour; and more rarely it is singularly pale. The epithelium sometimes separates, and an oozing of blood takes place from the raw surface. In other cases, the surface becomes hard, dry and horny; while in a third set, it is coated with a soft and moist crust of a white or yellowish colour. A cream-like coating is observed in a few examples of the disease.

These various states of the tongue may show themselves at the commencement, or at an advanced stage of the malady; or they may become apparent, only when it is subsiding. It is not to be forgotten that all of them may be present in cases, in which no gastro-enteritis is, or has been present. (From these details, it must be evident, that the appearances of the tongue are very fallacious, as a means of diagnosis.—*Rev.*)

The general or constitutional symptoms of inflammation of the digestive canal are especially interesting, as evidences of the very remarkable sympathy which exists between every part of the body and this great visceral apparatus. The nervous and circulating systems become almost immediately affected. In the earliest stage, the pulse very generally is full and frequent, the skin hot and dry, and the general symptoms are those of synochus. This feverish state has either a continued, remittent, or even an intermittent type, and it may be inflammatory, mucous, bilious, or putrid in its prevailing characters. These different forms of the disease are less dependent on the intensity of the gastro-intestinal inflammation, than on the conditions of the nervous and sanguineous systems of the patient, just before and at the period of his illness. Now, although these various types of fever are very often accompanied, perhaps we may even say occasioned, by that diseased state of the mucous lining of the stomach and bowels which we have been describing, it is ever to be remembered, that they may exist without any such local disease. (This paragraph announces the dissent of Andral from the exclusiveness of the Broussaian and other such creeds.—*Rev.)*

It cannot, therefore, be too urgently impressed on the mind of the young physician, that no disease requires a more minutely patient examination, to arrive at correctness of diagnosis and success of treatment, than gastro-enteritis; and none more imperatively demands the exercise of an unbiassed mind, which will enquire, and think, and determine for itself, from actually appreciable facts, and not be led along by the descriptions of authors however distinguished. Let it not be forgotten, that gastro-enteritis may be present, even to an alarming degree, without being indicated by any distinct symptoms of local distress; and that, perhaps, the only visible sign of its presence may be a febrile movement, sometimes simple, in other cases accompanied with divers disturbances of the nervous system. Such ataxic and adynamic forms of the disease are, we repeat, influenced less by the intensity of the local mischief, than by the conditions "d'innervation et d'hematose" which may be existing at the period of invasion.

As we might, à priôri, have supposed, the general character or type of the constitutional disturbance is greatly influenced by the age of the patients, as well as by their individual temperaments, the season of the year, the climate, &c. In infants and young children, it frequently assumes a convulsive or a comatose form; in old people, it is very apt to become alarmingly adynamic, and have a tendency to extreme apathy or exhaustion. When this tendency shews itself in the early stages of the disease, the diagnosis may be very obscure and uncertain, and the physician be led to suppose the case to be one of primary encephalitis, or meningitis.

As intimately connected with gastro-enteritis, in many of its essential phenomena, and necessary as its study is for the elucidation of abdominal disease, the subject of "follicular enteritis," or inflammation of the mucous follicles which are scattered over the inner surface of the intestinal tube, naturally follows for our investigation. It is well known that these follicles, or minute glands, are variously arranged in different portions of the bowels. In some places they are single, scattered, and separated the one from the other; whereas, at other points of the canal, especially along the tract of the ileum, they are assembled together in groups. The former have been called "glandulæ solitariæ, vel Brunneri," the latter, "glandulæ aggregatæ, vel Peyeri." It has been only within the last twenty years, that the attention of physicians has been studiously directed to the pathology of these glands, and to the diseased states of the system, which seem to be induced by inflammation, and other morbid processes affecting their structure.

MM. Petit and Serres described these lesions in 1811, and denominated the accompanying constitutional disease "entero-mesenteric fever." MM. Chomel and Louis subsequently described it under the old name, "typhoid fever;" M. Bretonneau has more recently called it "dothinenteritis;" Broussais has applied the same name to it, as to the disease which we have already alluded to, viz. "gastro-enteritis;" Bouilland prefers the title, "entero-mesenterite typhoide:" in the first vol. of my Clinique Médicale, I employed the term "exantheme intestinal," but now I think that the term "follicular enteritis" is more explicit and appropriate. The anatomical characters of this disease ought to be thoroughly understood by every practical physician. It is primarily and essentially an affection of the mucous follicles, or glands of the bowels. The mucous coat itself is involved only secondarily, or from the contiguity of the morbid glands; and, indeed, the circumscribed limits of the numerous inflamed and ulcerated points form a very remarkable feature of the pathology of the disease. The portions of the intestinal tube most frequently and most seriously affected, are the lower extremity of the ileum, the ileo-cæcal valve, the cæcum itself, and some parts of the colon. The upper divisions of the small intestines are much more rarely diseased, and, in the majority of cases, the stomach does not exhibit any abnormal appearances: "celui-ci est sain dans le plus gland nombre des cas—on n'y a jamais observé de cryptes isolés." In the early stage of the disease, the isolated follicles are seen as small papulæ, raised above the level of the mucous surface. At first they are of a lively red colour; but, during the course of the disease, they become of a brownish hue: sometimes these papulæ quickly subside, and even disappear altogether; at other times they are more stationary, and pass to a state of chronic enlargement. When they become ulcerated, the ulceration commences at the summits of the papulæ, and extends gradually deeper and deeper to their bases. The number of these papulæ is very various in different cases—they may be either few and scattered, or more numerous and confluent, just as we find that the eruption on the skin in small-pox is sometimes distinct, and at other times is confluent. The resemblance of the intestinal to the cuticular eruption is more striking, when the follicles of the former become ulcerated, for then they very frequently exhibit obvious umbilicated depressions at their summits. When the ulceration has proceeded farther, the entire substance of the enlarged follicles may be utterly destroyed, and, as it were, eaten clean away.

But it is chiefly among the aggregate or clustered follicles, the glandulæ Peyeri, that we shall find the most obvious and unexceptionable evidences of the disease at present under consideration. The groups of these follicles, which are by no means very obvious in the healthy state, present, during the course of a typhoid affection, the appearance of ovular or rounded patches, of a bright red or brownish colour. The prominence of these patches is attributable, not solely to the enlargement of the follicles, but also to the thickening of the surrounding submucous cellular texture. Their surface is uneven, granular, or somewhat honeycombed. When the disease terminates by resolution, the papulæ subside, and lose their bright colour; after the lapse of some time, the affected surface exhibits numerous dark dots, and hence it has been described as resembling a recently-shaven beard. When, on the other hand, the diseased process advances, the papulæ become ulcerated, the erosions become deeper and more extensive, until the whole extent of the clustered patches becomes one ulcerated surface. In some unfavourable cases, the patches are seized with gangrene; the eschars, on becoming detached, leave deeply-seated excavations, the edges of which are formed by the mucous membrane, and the bases by the submucous cellular, or even by the muscular tissue of the bowel. It is not of unfrequent occurrence, that the ulceration, at some points, proceeds through the entire thickness of the gut, and thus gives rise to perforations, through which the intestinal contents may escape into the abdominal cavity. Occasionally, considerable branches of the mesenteric bloodvessels have been exposed, and even opened by these ulcerations, and patients have died from the alarming hæmorrhage which has ensued. When the ulcers heal, their edges and bases become gradually smoother and more level—the surfaces become covered with a thin pellicle, and this pellicle assumes, in course of time, all the appearances of a genuine rudimentary mucous membrane, and, like the mucous coat of the bronchi and frontal sinuses, without any villosities. It is not unfrequent to observe a puckering of the surface, at the places where the ulcers have once existed. The number of the ulcerated intestinal patches is very different in different cases ; sometimes they do not exceed two, three, or four in number ; whereas, in other cases, they are greatly more numerous. As a general remark, it may with truth be asserted, that the gravity of the symptoms of the typhus fever is proportionate, cæteris paribus, to the number and extent of these lesions. The mucous surface, in the intervals between the diseased patches, may be either sound, or may exhibit traces of disease, varying from a simple inflammatory redness, to a thorough "ramollissement" of its texture, or to distinct and deep corroding ulceration. The redness, and even the softening, may extend as high up as the stomach. Even the œsophagus and pharynx have been found "offrir plus souvent des ulcerations dans cette maladie, que dans toute autre affection."

It is a question of much importance, to determine whether the symptoms of typhus fever are attributable to the follicular disease, or whether this disease is to be considered as only coincident, or simultaneous—and, in short, whether it is one of the effects, or whether it is the cause of the fever. Have we data to shew at what period of a typhus case the follicular changes are first appreciable ? Perhaps not ; for we know of no example, where death occurred before the fifth day from the invasion of the fever. One such case, indeed, has been reported by M. Bretonneau, and in this case he found a distinct intestinal eruption. Andral has seen it in patients who have died on the sixth day; and Chomel and Louis not earlier than on the seventh and eighth days. The question must, therefore, be considered as still open to discussion. Another practical question, of almost equal importance, is whether genuine follicular enteritis ever exists, without being accompanied by the presence of typhoid symptoms.

"A cette question (says Andral), on peut hardiment repondre par la negative." Patches of ulceration on the mucous surface of the bowels have indeed been discovered, in the bodies of small-pox and scarlet-fever patients ; but this is an argument rather in favour of, than discountenancing, the idea of the connexion of the morbid lesion with the existence of typhus fever. In the epidemic cholera also, it is well known that many authors contend, that the follicular disease of the bowels is of invariable, and even of necessary occurrence ; but, independently of the admitted fact, that the diseased patches which were observed after death from cholera, did not exhibit all the genuine appearances of "follicular enteritis," it may be remembered that the cholera did, in truth, present many of the strongest features of a most malignant typhus. The follicular ulcerations, frequently found in the bodies of phthisical patients, are obviously very different from the diseased changes which we have described as coincident with typhus.

Associated very generally with the genuine follicular ulcerations, are certain morbid states of the mesenteric glands and lymphatics. These are found in a hard, swollen, and inflamed condition, and not unfrequently they are converted into suppurating cysts, full of matter. The spleen is frequently much enlarged at the same time, even to double or triple its natural size. More rarely it is reduced to a pappy consistence, without any increase of bulk. The lungs, too, in many cases of follicular enteritis undergo serious morbid changes, varying indeed in gravity, from simply increased vascularity to complete hepatization, or to ramollissement of their structure. Perhaps no set of organs, and no portion of the system, is more frequently involved in diseased action, by the existence of fever depending upon ulceration of the intestinal glands, than the blood-vessels, and more especially their contents. Of late years, the attention of pathologists has been more studiously directed to the examination of the blood in this and other maladies, than it had been since the downfall of the ancient humoral doctrines. Already much curious and important information has been derived from such enquiries.

With respect to follicular enteritis, it is still a disputed point whether the morbid alterations of the blood (amounting sometimes to a very copious admixture of purulent matter) are always consecutive to the disease of the bowels, or whether it is not frequently coexistent with, or even antecedent to, the earliest occurrence of this disease. According to one set of physicians, the lesion of the intestinal follicles is the primitive mischief; it is the origin and point of departure to all the disturbances of the nervous and of the circulatory systems. But while they contend for this connexion and dependence of the constitutional symptoms on the local disease, they are quite willing to admit that, when these symptoms have been once induced, they may persist, as it were, spontaneously, and become more and more serious.

The intestinal disease may be said to act in the production of the nervous disturbance a similar part to what the wound does in a case of traumatic tetanus. The wound is certainly the primary cause of the tetanus; but the wound, it is well known, may heal, and yet the tetanus may continue and prove fatal.

In like manner the local lesion may act, in cases of the follicular disease of the intestines.

In opposition to these views, it is maintained by other physicians that the local mischief is the effect of the previously-established constitutional disease; that it is therefore a consecutive lesion, and ought to be regarded only as one, among others, of the phenomena of typhus fever; just in the same manner as the cutaneous eruption of a genuine exanthematous fever is to be viewed not as the cause, but merely as a feature or symptom of the general malady.

Whichever of those doctrines we receive, and it is certainly not easy to determine which of the two is the more correct, let it never be forgotten by the practical physician, that when once the system becomes involved in the constitutional disease, the general symptoms most commonly overpower, as it were, and thus mask and conceal the symptoms of the local distress; so much so, that unless a most rigid and minute examination of the patient be invariably instituted, the very existence of any intestinal disease may not even be suspected, and the case will be apt to be regarded as one of a fever, which is strictly and essentially constitutional, or appertaining to the whole system, unmodified by any local malady.

The etiology of follicular enteritis suggests some questions of very difficult solution. Why the peccant matter or offending cause should affect the glands of the intestinal canal, in preference to other parts of the body, we cannot form even a rational conjecture; and we must be therefore satisfied with the simple knowledge and affirmation that it is so.

When any acrid or irritating substance is introduced into the stomach and bowels, we can readily understand how an inflammatory action of their lining membrane is induced; but such is not the case, as far as we are aware, in the majority of cases of that follicular disease, which we are now considering. We are therefore obliged, in our ignorance of the more immediate causes of the morbid process, to conjecture that there is some specific agent, which determines its development in the intestinal gland; just in the same manner that in the exanthemata there is a specific determination to the skin. In both cases there is a *something* which is peculiar, and sui generis, in its origin and mode of operation. We know that, although we irritate the skin in all sorts of ways, and with every kind of stimulant, we cannot induce a variolous, scarlatinous, or rubeolous eruption: and so it is with follicular enteritis; no irritants received into the stomach and bowels will occasion those morbid changes of the intestinal follicles, which are found in the bodies of those who die of typhus fever. It has been indeed affirmed by some experimenters, that by injecting putrid matters into the veins of animals, the symptoms of genuine typhus have been induced, and that, on examining the bodies of such animals as have died in consequence of the operation, the intestinal follicles have been found inflamed and ulcerated. But these experiments require to be repeated before we can admit the correctness of the conclusions which have been drawn from them; and it may be worthy of notice, that in the animals on which the trials have hitherto been made, viz. horses and dogs, the intestinal follicles, in a state of health, are proportionably much more distinctly developed than in the human subject; and it is therefore possible, that the normal development may have been sometimes mistaken by pathologists for morbid enlargement. In tracing the history of follicular enteritis, all attentive observers have been struck with the especial liability to the disease, which those who have recently changed their residence from the country to a large city, seem to encounter. By far the greater number of the patients who are admitted with fever into the public hospitals of Paris occurs among those strangers who have recently arrived in the metropolis. It is generally during the first two years, and more especially from the sixth to the fifteenth month of their residence, that the disease is apt to invade them. We need not say that the disease is always more prevalent and more malignant in the crowded than in the more open and airy parts of the town.

With respect to the contagion of the fever, which accompanies follicular enteritis, M. Andral uses these words: "I should not hesitate to answer this question in the negative if I were to found my judgment on the results of my own observations, excluding the reports of others. I have not met with a single case, which unequivocally argued in favour of contagion." This distinguished physician, however, admits the high authority which countenances the opposite opinion. He alludes, in terms of particular respect, to the names of MM. Bretonneau and Gendrin, who have described several epidemics of the disease (denominated by them dothinenteritis) with more than usual zeal and accuracy, and who have been led to believe in its occasional contagiousness. "Il faut savoir douter," says M. Andral, with much truth and liberality of sentiment, "là où il y a doute." One of the most interesting features in the history of this disease consists in the very great difference in the liability to its attack of different ages. It is most frequent in persons from 18 to 30 years of age; it is comparatively rare among persons above forty; and still more so among those who have passed their fiftieth year. Not a single authentic case of genuine follicular enteritis in a patient upwards of 60 years, has been witnessed by M. Andral. It is sufficiently common in persons of from fifteen to eighteen years of age; but it is certainly of rare occurrence in younger patients. Some physicians have asserted that genuine typhus is never observed in very early youth; but this assertion must be received with hesitation.

Dr. Constant, who for some years past has devoted much attention to the practice of the Hôpital des Enfans Malades, communicated to M. Andral the results of the observations he made there during a period of 22 months. Twenty cases of genuine typhus occurred during this period; and the following table shews the relative frequency of the disease at different ages.

Two of the patients were 4 years old.

| | |
|---|---|
| One | 7 |
| Two | 8 |
| Two | 9 |
| One | 10 |
| Four | 11 |
| Four | 12 |
| Two | 13 |
| Two | 14 |

*Lancette Française.*

### On Methodic Compression, as a Remedial Agent in Dropsies.

The most obvious and useful effects of compression, applied over a considerable extent of surface, are to diminish the distention of the blood-vessels, to promote the action of the absorbents, and to give support to parts which have become relaxed and enfeebled.

These effects are very apparent in the treatment of elephantiasis of the leg, by a steady and methodic compression of the limb, exercised by means of strips of adhesive plaster, and by rollers applied regularly round its whole extent from the toes upwards. Certain cutaneous phlegmasiæ, especially those forms in which the subjacent cellular texture becomes involved, have been successfully treated in the same manner. M. Bretonneau, in his thesis on this subject, published in 1815, has narrated several cases, to prove its good effects.

It is, however, more particularly in œdematous and other dropsical affections, that compression may be used with the most decided advantage. The following are some, from a multitude of examples, in which Dr. Bricheteau has had recourse to it.

Case 1.—*Œdematous Swelling of the Right Lower Extremity.* A woman, 52 years of age, whose health had been hitherto very good, observed, two months previous to her admission into the hospital, that the right foot and leg had become considerably swollen, so as to incommode her greatly in walking. In the course of a fortnight afterwards, the swelling extended, and reached as high as the top of the thigh. The catamenia had been absent for four months. She was ordered warm-baths, cooling evaporating lotions to the limb, and blisters on the calf of the leg.

On the 12th of April, when received into the hospital St. Antoine, the thigh, leg, and foot were found to be much swollen, the skin being red, distended, and shining, and when pressed with the finger, it exhibited only "une empreinte fugace." The course of the large blood-vessels was unattended with pain or engorgement: all movements of the limb were constrained and uneasy. At the knee, the circumference of the limb exceeded that of the other one by eight or nine inches. The inguinal glands were not swollen. The general health was unaffected.

M. Rayer, under whose care she was placed, ordered the patient to be bled, and to apply emollient fomentations to the affected parts. The tumefaction, however, continued to increase; and the patient began to experience pain along the course of the blood-vessels. Absolute rest was enjoined, and the use of the fomentations was continued. On the 15th, scarifications were made at different parts of the thigh, and on the dorsum pedis. A small quantity only of serosity mixed with blood escaped from the punctures. On the 27th, the punctures being healed, compression by means of a roller applied from the toes to the middle of the thigh was commenced. During the following three weeks the swelling of the limb very sensibly abated.

On the 21st of May, in consequence of general malaise, head-ache, and an eruption of papulæ on different parts of the surface, the patient was bled twice from the arm;—the blood was sizy.

The limb continued to decrease in volume, but it was still hard to the finger, and the skin was tense and firm. In the first week of July, the same means having been persevered in, the patient began to walk about a little; and on the 19th of this month she was discharged, although the limb remained still somewhat swollen, and the subcutaneous cellular tissue rather indurated.

[*Remark.* The recovery was certainly very tedious, and after all not complete. The constitutional treatment was probably not pursued with the requisite diligence—*Rev.*]

Case 2.—*Ascites cured by Compression.* In 1824, M. Godelle published in the Nouvelle Bibliotheque Medicale the following report of a case in which compression was employed with decided advantage.

A youth, 18 years of age, of a feeble sickly constitution, was admitted on the 5th of July into the hospital at Soissons, in consequence of diarrhœa, colicky pains of the abdomen, &c. The skin was hot and parched, the pulse was small, contracted, and sharp; the urine was scanty, and of a high colour; and an indistinct fluctuation within the abdomen was perceptible.

Leeches were applied on the epigastric and hypochondriac regions; oily liniments and emollient fomentations were then used; and mucilaginous nitrous drinks and a low diet were prescribed. These remedies were, with occasional slight changes, continued for a fortnight. Although the abdominal effusion did not diminish, the patient left the hospital; but he soon returned in a worse condition than before. He was harassed with a dry frequent cough; the abdomen was much distended and painful; the urine was scanty and red; the skin was dry; the pulse was small and quick; and for some days past he had been teased with diarrhœa. Recourse was again had to the use of oily embrocations rubbed on the abdomen; and small doses of digitalis were added to the mucilaginous drinks. The swelling of the abdomen however progressively increased, so as seriously to impede the breathing, and even to threaten suffocation. M. Godelle being anxious to try the effects of compression, before resorting to the operation of paracentesis, ordered that a roller should be applied firmly and regularly round the abdomen. No sooner was this done, than the respiration became more easy and free. As the roller became displaced by the movements of the body, a broad belt, laced like a lady's corset, was substituted for it.

"Cinque jours suffirent pour ramener le ventre à son volume ordinaire et faire disparaitre le liquide epanché." As the absorption of the effused fluid proceeded, the compression was steadily increased, and small doses of digitalis were administered at the same time.

CASE 3.—*Ascites of six months' standing.* A young woman had been affected with ascites for six months before applying for medical relief. The effusion had come on very gradually, and had been unattended with pain, or with any constitutional disturbance. She attributed her illness to exposure to cold, and to residing in a damp house.

The abdominal effusion increased so much within the three first months, that the breathing became much impeded, and her general health began to suffer. She was admitted into the hospital on the 23d of January. The fluctuation could be very easily recognised. No signs of any visceral disease were present. The symptoms were relieved for a time by the use of diuretics, squill, and digitalis, and of drastic purgatives; but the symptoms returned with greater severity than before, and it was then deemed quite necessary to withdraw the fluid by tapping. After the operation, active doses of jalap, combined with nitre, were administered, and had the effect of increasing the urinary discharge so much, that the abdominal effusion was kept under. But this favorable condition was merely temporary, and the ascitic symptoms speedily became as aggravated as ever. Recourse was then had to "compression methodique," by means of a bandage or belt, well fitted to the whole surface of the abdomen, and laced like the female corset, so that it could be readily tightened or slackened according to circumstances.

The pressure and support were thus uniform at every part. "Les urines ne tarderent pas à couler plus abondamment, et eprouverent en meme temps un changement favorable dans leur couleur et leur densité." The size of the abdomen gradually decreased, and in the course of a month, all sense of fluctuation had ceased.

CASE 4.—*Ascites.—Compression continued for eight months.* A man, nearly 60 years of age applied to Dr. Bricheteau in consequence of ascites, which had existed for several months. His general health was very indifferent, and his appearance indicated a strong tendency to universal dropsy, the features being pale and bloated, and the skin being lax and cold. He suffered occasionally from epigastric pains, vomiting and dyspepsia. On examining the abdomen, some degree of induration in the epigastrium and right hypochondrium was distinctly perceptible.

Leeches and afterwards blisters therefore were ordered to be applied over this region, and various diuretic medicines were administered. Recourse was then had to the continued use of graduated and methodic compression by means of a laced belt, which firmly and equally embraced every point of the abdomen, from the chest to the haunches.

The belt was never laid aside, night or day, during the following eight months, although for some time previous, all signs of abdominal effusion had vanished. Dr. B. attended this patient subsequently for another complaint; but there was no return of the ascites.

Case 5.—*Ascites—Ague.—Cure in three months.* A woman, aged 37, was admitted into the hospital in consequence of an attack of ague. The use of the sulphate of quinine and other appropriate remedies speedily removed this disorder; but then symptoms of anasarca and ascites began to shew themselves. The abdominal effusion increased so much, that a considerable degree of dyspnœa was induced, especially when the patient lay down, or took any active exercise. Diuretics and purgatives failed to afford much relief; and paracentesis was therefore performed. Upwards of twelve pints of fluid were withdrawn. The effusion so quickly returned, that the operation was again necessary in the course of three weeks. For three or four days, the fluid continued to ooze copiously from the puncture made by the trocar. Immediately after the performance of the second operation, the abdomen was equally and firmly compressed by means of a roller applied with much exactness and care, and "quelques boissons diuretiques et des purgatifs" were administered. The use of the roller was continued steadily for three months, at which time the patient was pronounced entirely cured. Four months afterwards this woman returned to the hospital, in consequence of an attack of acute gastro-enteritis, which ultimately proved fatal. On dissection, the abdomen was found quite free from any effusion. There were some adhesions of the peritoneum at several points; but there was no decided disease of any part, except of the mucous coats of the stomach and bowels, which exhibited the traces of active inflammation.

In the 40th volume of the Annali Universali a case illustrating the curative effects of steady compression of the abdomen in ascites is recorded. The disease had supervened upon an attack of peritonitis, and had existed for several months, before the patient applied for medical assistance. She suffered from derangement of the digestive functions, and from general feverishness—the urine was scanty and turbid; and there was considerable emaciation of the whole frame. The distention of the abdomen was so great, that no accurate examination of the state of the viscera could be instituted. The usual remedial means having utterly failed, Dr. Speranza was induced to try the effects of graduated compression of the abdomen, by means of Dr. Monro's bandage. The flow of urine became speedily much increased, the patient passing sometimes as much as twelve pints daily. Under the use of tonic medicines and of a generous diet, a complete cure was soon established.

Case 7.—*Ascites of four years' standing.* A woman, 40 years of age, had been affected with a dropsical enlargement of the abdomen for nearly four years, when she applied to Dr. B.

The disease had come on very gradually, and it caused but little inconvenience, except from the bulk of the swelling. Menstruation had been regular, and the other functions of the body were but little disturbed.

Dr. B. recommended that the fluid should be drawn off by tapping; but she objected to this operation. Diuretics and purgatives were used for some time with little benefit; and then she submitted to paracentesis. Twenty pints of a limpid serum were drawn off. No traces of diseased viscera could be detected on the most careful examination, which was most satisfactorily made, in consequence of the extreme thinness of the abdominal parietes, when relieved from their distention. The use of the laced belt was steadily persevered in for several months after the operation; and by this simple means alone, without the assistance of any other remedy, a perfect cure was effected. The patient continued to wear the belt for many months after all traces of fluctuation had disappeared.

The curative effects of methodic compression in dropsical affections of the abdomen are sufficiently illustrated by the preceding reports. Of late years, its use has been extended to the treatment of chronic hydrocephalus by certain American and English physicians. Dr. Glover, of South Carolina, having unsuccessfully tried all the usual remedies in a case of a young child affected with this form of the disease, resolved to draw off the encephalic fluid by a paracentesis of the cranium. The circumference of the head measured two feet; the sutures were very open and wide; the fluctuation was distinctly perceptible; there was strabismus, but the general health of the patient was very satisfactory. The puncture was made in the course of the squamous suture. A pint of serum was drawn off through the canula. The falling together of the integuments and "le jeu des pieces osseuses les unes sur les autres" induced Dr. G. to withdraw no more at the time. He encircled the head with a bandage, applied several times round, in order to maintain a steady and equable compression on every part. No disagreeable symptoms ensued. The urinary secretion became very sensibly increased in quantity. Two days afterwards, another pint of serum was withdrawn by the same orifice, without any disagreeable result, and the bandage was immediately re-applied. After other four days had elapsed, a new puncture was made, to give exit to a fresh accumulation of fluid; and this time, not less than three pounds were drawn off. During the following ten days, the infant appeared to improve gradually, under the influence of the methodic compression of the head. It gained flesh; the cranial bones became approximated to each other; the urine flowed copiously; and the strabismus ceased.

Notwithstanding these favorable changes, a re-accumulation of the fluid required the performance of another operation; the puncture was made in the course of the coronal suture, and a pint of fluid escaped. For upwards of a month, a decided amendment followed this operation; but it was subsequently found necessary to repeat it twice. The child died on the eighth day after the last operation. On dissection, it was found that the substance of the brain was almost completely wasted away, and that there were three pints at least of fluid within the cranial cavity. In this case of Dr. Glover, 156 ounces in all were withdrawn at eight different operations, in the course of four months. (New York Med. Repos. vol. 4, 1819.) Unfortunately the results of the majority of the cases hitherto recorded, where paracentesis of the cranium has been performed have been the same, as in Dr. G's case. At one time it was supposed, that the water in chronic hydrocephalus was always collected in the sac of the arachnoid, and not within the ventricles, and consequently that the cerebral substance need not be wounded in the operation; but this is now known to be a mistake.

Dr. Graefe has narrated the particulars of one successful case in his practice. The infant was born hydro-cephalic. When four months old the head was punctured, and the operation was subsequently repeated eleven times in the course of six months. After the last puncture, the sutures closed. At the age of two years and a half, the child was shown to the Medico-Chirurgical Society of Berlin. Mr. Russell of Edinburgh also has recorded a successful case; and we are informed that Dr. Conquest of London has succeeded in four out of nine cases operated on by him. M. Breschet is therefore not justified in condemning so peremptorily the operation, as we observe that he has done in the following passage from the article Hydrocephale. Dict. de Medecine, vol. ii.; "cette evacuation amene la mort plus ou moins promptement, et dans tout desespoir de cause, rien ne peut excuser la pratique d'une operation qui accelere la fin des malades, et qui leur fait suffrir des douleurs inutiles." Although we caution our readers not to anticipate great success from the operation, yet under certain circumstances, and in judicious hands, it may very properly be resorted to.

The employment of compression only (without an operation), has been recommended by Sir Gilbert Blane and others; and as this remedy is unattended with any danger, and its employment may be suspended at any time, when it appears to be unadviseable, it will be more generally adopted, than the operation of paracentesis of the head.

The compression may be exercised, by means of a roller only, applied several times round the head, or by using encircling strips of plaster at the same time. In the first case, related by Blane, the child was sixteen months old; the head had been very large from its birth, and the anterior fontanelle was very large. There was also a curvature of its spine. For several months, the child had been almost constantly in a state of stupor and oppression; the pupils were dilated; and from its cries and groans, and from applying its hand frequently to the head, it seemed that the little patient suffered much inward pain. A roller was applied with moderate tightness round the head, and an active purgative was given occasionally. In the course of three months, all the cephalic symptoms had ceased, and the health of the infant was completely restored.

The second case occurred in an infant, three years old; the head was very large, and the anterior fontanelle still open. The same treatment, as in the former example, was adopted; and with most marked advantage. This practice has been followed by other physicians with occasional success. Fortunately it is quite safe, and if judiciously employed, cannot produce any evil. "La compression" says Dr. Bricheteau "loin d'avoir causé des accidens, a produit du soulagement, et ralenti l'epanchement du liquide dans la cavité crânienne; de plus elle a augmentée la secretion urinaire; phenomene digne d'etre medité, surtout parcequ'on en observe un tout-a-fait semblable lorsqu' on comprime le ventre chez les individus atteints d'ascite."

Compression acts in the cure of dropsy, partly by preventing the further exhalation of fluid, and partly by exciting the absorption of that already deposited. The pressure exercised by the bandage is communicated to the contained fluid; this in its turn presses on the exhalant surface, and mechanically prevents the afflux of any more serosity.

Thus the effusion necessarily remains stationary, since it is not possible that any fresh fluid can be admitted into a cavity which is already not distensible; "d'ou une retropulsion de la serosité separée du sang, retropulsion qui se communiquant de proche en proche dans les canaux pleins, doit apporter une modification quelconque dans le mecanisme de la nutrition." This modification must be somewhat analagous with the effect which compression on any bloodvessels has in throwing back the blood into those vessels, which are either above, or below the point compressed, according as the vessels are arterial or venous. When any effused fluid has been dispersed, it is customary to say, that the absorbents have "repompé" it; but perhaps it might be more correct to attribute the dispersion to a sort of imbibition, the mechanism of which is analogous to that of compression when applied to any of the bloodvessels. No one doubts in the present day, that, by means of imbibition, the living tissues are susceptible of being penetrated and traversed by the animal fluids, without the intervention of any exhalant, and absorbent process. There are cases indeed, in which the supposition of any imbibition is not easily admissible; as, for example, when compression causes the disappearance of an albuminous mass floating in a fluid; and also when the compression is exercised on a cavity which is surrounded with hard unyielding structures, as in the case of the joints. M. Godille has attributed the remedial effects of compression in the treatment of dropsies, to an increased activity of the venous absorption, induced, he says, by the retardation of the blood in the abdominal aorta, and by the pushing forward (refoulement) of the abdominal venous blood, and its accelerated return into the vena cava. This explanation, which seems to us not very perspicuous, is rather contradicted by the effects of copious bleedings; for these, it cannot be denied, render the arterial and venous circulations more free and rapid, and at the same time "activent d'une maniere notable l'absorption des fluides epanches."

Another effect of continued compression, is the production of adhesions between the different abdominal viscera; adhesions which necessarily assist in opposing future effusions.

In concluding these remarks on compression as a remedy against dropsy, it is proper to observe, that in some patients it is inadmissible, in consequence of its inducing dyspnœa, "par un mechanisme qu'il est facile de comprendre."—*Clinique Medicale de l'Hopital Necker.*

---

### Treatment of Gonorrhœal Ophthalmia.

Almost all surgeons, of large experience in the diseases of the eye, admit that gonorrhœal ophthalmia is attributable generally, if not solely, to the inoculation of the poisonous virus, either directly from the urethra, or from an eye already affected with the disease. In the few cases, in which we cannot trace the operation of this cause, we may reasonably infer that the patient has accidentally and unknown to himself conveyed the matter to his eye: this he may have done during sleep, or when his attention was at other times unguarded. What confirms this view of the etiology of gonorrhœal ophthalmia, and establishes a very marked difference between it, and other forms of purulent ophthalmia, such as the catarrhal, is that in the majority of cases of the former, one eye, and that usually the right one, is involved, whereas, in the latter, both are almost always affected simultaneously. Beer, Cooper, Vetch, are high authorities for these assertions.

Now if the gonorrhœal species was owing to a metastasis, or sympathetic translation of the morbid action from the urethra to the eye, we might surely expect that both organs would suffer at the same time. Some surgeons have objected to the view, which we have taken, on the ground that if gonorrhœal ophthalmia was generally induced by the direct application of the virus to the organ, the disease would be infinitely more frequent, as it is well known, that most patients observe few, or no precautions against its occurrence. But then, not to allude to the merely conjectural character of the objection, let it be remembered, that if the rarity of the disease, compared with the extreme frequency of the inducing cause, is to be received as an argument against the idea of a direct inoculation, the same argument applies to the other hypothesis, which assumes, that the disease is of a metastatic or mere sympathetic origin: Why should the supposed metastasis, or morbid sympathy not occur, more frequently than it does? The assertions of the most experienced surgeons all lead to the same conclusion. Sanson and Mr. Lawrence distinctly affirm that they have not met with a single case of gonorrhœal ophthalmia, when the urethral discharge had altogether ceased. M. Sanson says, that in the few cases, in which the discharge had apparently ceased, he could always satisfy himself of the actual existence of it, by squeezing the extremity of the penis. In reference to the supposed metastasis of the poisonous matter, it deserves to be remembered, that the urethral discharge is not only not always suppressed, but that in some cases it even becomes more profuse, after the ophthalmia is fairly established. Another important fact, is that genuine gonorrhœal ophthalmia is very rarely met with in females. Beer and Lawrence tell us that they have never seen a case of it in women. M. Dupuytren has indeed expressed an opposite opinion; but, in spite of his high authority, we are compelled to assert that his opinion on this subject is not quite correct. The causes of the extreme rarity of the disease in females is probably to be sought for in the admission, that they " ont beaucoup moins que les hommes l'habitude de porter les doigts à leurs organes genitaux, et que chez elles on rencontre moins frequement ces ophthalmies chroniques que nous avons vues predisposant si fortement, à contracter l'ophthalmie blennorhagique." If this explanation be correct, and we believe it to be so, another argument against the metastatic origin of the disease is furnished to our consideration. As the chief object of these remarks is to direct the attention of our readers to the treatment of gonorrhœal ophthalmia, we shall now without further delay proceed to glance at the comparative merits of the very different modes of cure, which have been proposed. Perhaps no disease demands greater promptitude and decision of practice, than this form of ophthalmia. The organ may be, and very often is, irretrievably damaged in the course of four and twenty hours, the cornea having become opaque and ulcerated, and even the internal structures of the eyeball having already proceeded to disorganization. This extreme rapidity of the mischief, coupled with the great discrepancy of the most distinguished oculists as to the most efficient means of arresting its progress, is apt to confuse and bewilder the younger practitioner, and to render his practice unsatisfactory to himself, and probably most unfortunate for his patient. The most vigorous antiphlogistic treatment considered by some to afford the only rational hopes of saving the sight, is confessedly very often, or rather almost always, wholly ineffectual, unless enforced from the commencement of the disease, before the suppurative stage has become established. Lawrence, who, it is well known, is a most resolute advocate of depletion, admits, that the loss of vision is almost inevitable, if the surgeon be not called at the setting in of the first stage. We shall not argue this therapeutic question on any speculative grounds, as to the nature, or character of the inflammation in gonorrhœal ophthalmia, but shall merely glance at the results of the antiphlogistic practice, as recorded in the writings of some of its most distinguished supporters. Lawrence has detailed twenty-four cases in all of gonorrhœal ophthalmia. In fourteen of these, the disease was of the most acute form; nine of the patients lost the sight of the affected organ, in consequence, either of suppuration and ulceration, or of induced opacity of the cornea. In the other five cases, the sight, although impaired, was saved ; in all of them, the cornea was left partially opaque, and in three, the anterior surface of the iris had contracted adhesion to the inner surface of the cornea. The following cases will give some idea of the *success* of the antiphlogistic treatment. A youth, 20 years of age, had inadvertently wiped his eyes, with a cloth, which was stained with some urethral discharge. When he applied for relief, the cornea of the left eye presented a whitish-coloured eschar, which covered nearly a fourth part of its surface; the right eye was not affected at this time. In the course of forty-eight hours, eighty ozs. of blood were drawn by the lancet and cupping-glasses, and 170 leeches applied, not to mention the use of blisters, drastic purges and antimonials. On the fifth day, an ulcer appeared on the right cornea, and the left cornea was somewhat staphylomatous. Five weeks after this date, it is reported that the ulcer of the right eye was not quite healed ; and the sight of the left eye was still very imperfect. In the second case, the depletion was carried to a greater extent. In the space of two days 154 ozs. of blood were drawn from the arm and temporal artery ; eighty leeches were applied; strong doses of calomel and saline purgatives were taken every two hours, and blisters were applied behind the ears and on the temples. On the ninth day, the hyaloid membrane protruded through an ulcerated opening of the cornea. A month after this date, the swelling of the cornea had not entirely disappeared; but the sight was but little affected.

A year and a half afterwards, the cornea exhibited an opaque spot, about a line in diameter, and to this spot, the iris was still adherent. These cases afford a fair specimen of the practice pursued by this eminent surgeon, and of the success, which has attended it in his hands. Indeed it may be fairly asserted that not one case of perfect and absolute cure has been recorded by him. Mr. Wardrop has been quite as bold, and apparently also, about as *fortunate* as his brother Anglois. He is candid enough to acknowledge that the only case of severe gonorrhœal ophthalmia, in which he effected a complete cure, was that of a young woman, who was bled from the arm, as often as blood could be obtained. She lost 170 ozs. in three days! The late Baron Dupuytren, convinced by unfortunate experience of the inefficacy, not to speak of the danger, of extreme depletion in this disease, renounced it in the later years of his practice, at least as the exclusive, or even as the principal means of cure; and the same remark is true of M. M. Sanson, Roux, &c. The opinions of these surgeons are the more valuable, because every one of them had at one period or other of their professional career, trusted to depletory measures in the treatment of gonorrhœal ophthalmy, on the supposition that the disease was purely inflammatory, and was therefore to be combated only by antiphlogistic remedies, proportioned in activity to the extent and severity of the morbid action. All of them have subsequently made a sacrifice of these opinions, by finding that the practical conclusions which are derivable from them are condemned by experience. In the present day very few of the French surgeons trust to the depletory practice as the chief implement of cure; and those that do, without exception confess that the results, which they have obtained in severe cases, have been very rarely satisfactory. It is not to be inferred, that bleeding is to be altogether discarded from the treatment of gonorrhœal ophthalmy—far from it. At the commencement of every case, one or even more large venæsections ought to be practised; but other active means must be resorted to at the same time, and of these perhaps the most powerfully efficacious is the local use of the nitrate of silver, especially with excision of a portion of the chemosed conjunctiva, according to the method now uniformly followed by M. Sanson at the Hôtel Dieu. We have already stated that this eminent oculist has been for some years past convinced of the "inefficacité absolue" of the antiphlogistic treatment, employed by itself. He however recommends that one very large bleeding should invariably precede the adoption of any local applications, or of the excision of the swollen conjunctiva. An impression is thereby made on the system, and the troublesome consequences, which would otherwise be apt to follow the performance of a severe operation on an organ already suffering from a most acute disease, are thus most effectually guarded against. If, contrary to his expectations, this first bleeding be followed by a decided relief to the symptoms, so as to lead him to believe that the disease may be more purely inflammatory than he had imagined, he does not hesitate to repeat it, and to apply at the same time an immense number of leeches in the neighbourhood of the suffering organ, watching the progress of the local disease most carefully all the time. When, on the other hand, no permanent or decided amelioration takes place (and unfortunately this is by far most frequently the case) he proceeds at once to perform the following operation on the affected eye. The eye-lids being separated from each other, and everted as much as possible, the projecting ocular conjunctiva is laid hold of with a pair of dissecting forceps, and excised by means of curved scissors. The excision ought to be as complete as possible, all the swollen membrane, as far as it can be reached, being firmly cut out. The discharge of blood is usually very copious. When it begins to subside, the eye is to be wiped clean of the blood and purulent discharge, and a stick of lunar caustic is then to be rubbed freely on the everted surfaces of both palpebræ. The parts should be bathed with tepid water, before replacing the palpebræ. The success of this treatment depends very much on the care which is taken to "cauteriser à un degré convenable la conjunctive palpebrale." For this purpose the nitrate is to be drawn slowly along the everted surfaces, pressing it chiefly on those points where the membrane is most swollen or granulated. If the membrane is in folds, the caustic should be passed, if possible, in between them, so that no part of the diseased surface escapes it operation. The pain caused by this treatment is, as might be supposed, excessively severe; but it does not remain long. The application of cold water to the eye will be found useful, not only as a sedative to the pain, but also as an astringent to the discharge.

However acute the suffering may be, and it is not unfrequently so great that the young practitioner is alarmed for the consequences, there is in truth no danger. The practice has now been employed at the Hôtel Dieu for some years, and there has not been a single case of mischief having occurred, although the nitrate has been used very freely and boldly. At first M. Sanson had always recourse to general bloodletting after the operation, to guard against the dreaded inflammation; but of late he has quite discontinued this precaution, unless the case required, for other reasons, the use of depletion. The rationale of the treatment by excision and cauterization, as adopted and recommended by M. Sanson, is sufficiently obvious. Part of the morbidly secreting tissue is removed, and the surface of another part is destroyed to a greater or less depth and extent, according to the freedom with which the nitrate has been employed. When the disease is arrested, the cut surfaces of the ocular conjunctiva speedily unite together by cicatrization; and as this takes place before the eschar has become detached from the palpebra, there is little or no danger of any adhesion being formed between the two opposite surfaces of the conjunctival membrane. We adduce two examples illustrative of M. Sanson's practice.

A Polish refugee, 27 years of age, of a strong healthy constitution, had contracted gonorrhœa a fortnight before his admission into the Hôtel Dieu. The gonorrhœal ophthalmia had, at this period, existed thirty-nine hours. The eyelids were prodigiously swollen, and very considerably everted. The ocular conjunctiva was so much chemosed, that the cornea could be seen only partially: it was still transparent, not softened, but bathed with the profuse purulent discharge, which irritated the cheek wherever it came in contact with it. The throbbing pain and intolerance of light were extreme. The patient was immediately bled to a large amount; 30 leeches were applied around the affected eye; and appropriate internal remedies were employed. On the following day, the 3d, the cornea was found to be somewhat opaque. The patient was much reduced; the leech-bites had never ceased bleeding for four-and-twenty hours. In the evening of this day, another venæsection to the amount of 25 ounces was practised, and a drastic purge was given. On the 4th, the eye was deemed irretrievably lost, the cornea being "ramollie et plissée;" next day it burst, and the iris protruded. At this time, although the patient was "excessivement faible, pale et decoloré," the other eye became affected with the disease in its most intense form. "Dans l'espace de quelques heures on voit se developper des granulations extremement nombreuses, lesquelles sont le siege d'un ecoulement très abondant de matières mucoso-purulentes."

To recur to depletory remedies was quite impracticable, considering the exhausted state of the patient's strength: "il aurait inevitablement succombé." M. Sanson, therefore, determined to practise the operation of excising the ocular conjunctiva, and of cauterizing the palpebral portion of the membrane. A small quantity of blood was then drawn from the temporal artery, and leeches were applied round the orbit. The intense pain, occasioned by the operation, subsided in the course of a few hours; and the patient then obtained some sleep. On the morrow, "l'amelioration fut tres sensible;" the tumefaction of the eyelids had subsided greatly, and the purulent secretion was "completement tarie." Next day, the condition of the eye was altogether much improved, and the pain had quite ceased. The patient could open the eyelids, and even perceive the objects around him. He gradually recovered the perfect sight of the right eye. The conjunctiva of the left one being still much swollen, and the purulent discharge from it continuing, M. Sanson excised the ocular, and cauterised the palpebral portion, as he had done to the other eye. A smart attack of ophthalmia was the consequence; but the discharge was quite stopped. The inflammation was subdued by appropriate means; and on the fourth day of April the patient was discharged completely cured.

*Case* 2. A man, 25 years of age, had been suffering from a neglected gonorrhœa, for a period of 15 months. On the first of October, 1834, he began to experience sharp pains in his left eye, which had become red and watery. On the following day, the ophthalmia had greatly increased; and the eyelids were glued together by a most viscid discharge. When received into the Hôtel Dieu on the 6th, both eyelids were excessively swollen: on separating them, a profuse quantity of muco-purulent matter flowed out: the ocular conjunctiva was so much chemosed, that it concealed the greater part of the cornea, which however seemed to be still quite transparent. The patient complained of intense pain in the ball of this eye; the pulse however was not much quickened, nor the pyrexia at all severe. Before his admission, he had been freely bled and purged, with but little relief to the symptoms. As the disease was rapidly making progress, M. Sanson excised the turgid ring or "bourrelet," which the chemosed ocular conjunctiva formed round the cornea, and cauterized freely the inner surface of both palpebræ with the nitrate of silver. The pain from the operation was most severe, and did not subside for several hours. A few drops of a collyrium, composed of two grains of sulphate of copper, two grains of Armenian bole, half a drachm of gum arabic, and ten drops of laudanum to four ounces of distilled water, were to be introduced between the eyelids occasionally. Orders were given, that if the swelling of the conjunctiva returned, a collyrium composed of six grains of nitrate of silver to four ounces of water, was to be used instead of the preceding one.

The puriform discharge ceased almost immediately, and the pain gradually diminished more and more. On the following day, the swelling of the conjunctiva had abated considerably, and the discharge was very trifling.

The employment of the injections produced much less pain than on the preceding day. On the 9th, the improvement was considerable. The whole of the cornea was now visible, and it was quite transparent. 11th. The swelling of the palpebral conjunctiva having increased somewhat, the collyrium with the nitrate of silver was used. 13th. All the swelling had disappeared, and the patient was pronounced cured.—*De l'Emploi de l'Excision, &c. dans l'Ophthalmie Blenorrhagique, par E. F. Julliard, Paris,* 1835.

The preceding observations make us acquainted with the mode of treatment recommended by M. Sanson in one of the most active and dangerous forms of purulent ophthalmy. Whether the gonorrhœal matter exerts any peculiar influence as a specific poison, or whether, as is more probable, it acts merely as an irritant to the mucous structure of the eye, the surgeon cannot fail to perceive that there is a close and intimate resemblance between the gonorrhœal and some other forms of the disease; and he is therefore legitimately induced to believe, that the treatment most successful in one form will, in all probability, be best adapted to the others. We are inclined to agree with M. Julliard, that the genuine gonorrhœal ophthalmy in most cases, and perhaps always, arises from the direct application of the urethral discharge to the eye, and seldom or never from its mere retrocession. That the discharge from the urethra is often much diminished, and sometimes even totally suspended, when the eye or eyes become affected, is quite reconcileable with what is observed in other diseases. The irritation of the one organ may act as a derivative or counter-stimulant to that of the other, without any metastasis of the diseased action. Independently of the arguments which our author has adduced in favour of the local origin of gonorrhœal ophthalmia, viz. its extreme rarity in females, the circumstance of it being very generally limited to one eye, and also of the urethral discharge not unfrequently continuing after the eye has become affected, the analogy of the exciting causes of other purulent ophthalmias, such as the Egyptian and infantile, adds much to its probability.

That a purulent ophthalmy may occur in infants, as well as in more advanced patients, from exposure to cold, or to strong light, from the application of various mechanical stimulants, and probably also from certain unknown epidemic influences, we have no intention to deny: but by far the most frequent cause is the direct application of the acrimonious vaginal discharge to the eyes of the infant during labour.

With respect to the cause of the Egyptian purulent ophthalmia, the testimony of Drs. Vetch and Edmonston, and of Mr. M'Gregor, leaves little doubt that the disease was generally propagated from one person to another by actual contact of the discharge. The late Mr. Ware, a high authority in ophthalmic surgery, has delivered his opinion "that this particular disorder is only communicable by absolute contact; that is, by the application of some part of the discharge, which issues either from the conjunctiva of the affected eye, or from some other membrane secreting a similar poison, to the eye of another person:" and in reference to the gonorrhœal species of purulent ophthalmy, he has stated, "that in the majority of adults, whom he has seen affected, if the disorder had not been produced by the application of morbid matter from a diseased eye, it could be traced to a connexion between the ophthalmy and disease of the urethra." So much for the etiology of purulent ophthalmia. We shall now proceed to the more important subject of the treatment of its more severe forms, and endeavour to shew that the practice of the most experienced surgeons coincides in principle with that recommended by M. Sanson in the gonorrhœal variety. This is the more necessary, as we believe that far too many of the surgeons of the present day have very vague and incorrect notions as to the plan which they ought to pursue in treating this and the other varieties of purulent ophthalmy.

The formidable appearance of the affected eye, the extreme rapidity of the disease, and its too often destructive effects, have led many surgeons to regard it as consisting in the most intense inflammation possible of the affected structures; and they have therefore supposed that the only chance of arresting the mischief, is by a corresponding activity in the use of antiphlogistic measures, and more especially of sanguineous depletion. Hence the advice of one author to take away not less than sixty ounces of blood at the first bleeding, and to repeat the operation, in a smaller quantity, at short intervals, until a decided amendment of the state of the eye is visible. But surely this practice is as unsound on theoretical grounds as it has proved to be unfortunate in its results. Inflammation of mucous tissues, especially when attended with an increased flow of their secretions (whether these be perverted in their qualities or not,) requires, as a general rule, less copious depletion than inflammation of any other texture.— Take, for example, some forms of cynanche or bronchitis, dysentery or gonorrhœa: in none of these diseases do we practise so large or so frequent venæsections as in pneumonia, peritonitis, &c.

The vehemence of the inflammatory action is never so great in the former cases; the mucous membranes have a tendency to relieve themselves of the hyperæmic state by an augmented flow of their accustomed secretions; and the conduct of a judicious physician is rather to be guided by Nature, than directly to oppose and thwart the course she points out.

Now if this principle be true of these diseases, it is equally so of the maladies of the eye. Inflammation of its fibrous tissues requires much more vigorous depletion than inflammation of its mucous membranes; and let it be remembered, that it is these membranes which are primarily and chiefly affected in all purulent ophthalmias. But, not to trust to mere theoretical objections, we shall now briefly glance at the course of treatment which has been found most useful in some of the most common forms of the disease: and first of the Egyptian ophthalmy, including that variety which prevailed to such an extent in the Royal Military Asylum at Chelsea, and in other places, about the beginning of the present century, and of which a very admirable account was published by the late Sir P. M'Gregor. Dr. Vetch recommended that one large bleeding should be practised at the commencement of the disease; but he cautions us against trusting to antiphlogistic remedies alone, however vigorously employed. The use of powerfully astringent and sedative applications to the eye is much insisted upon; and of these he gave the preference to the undiluted liquor plumbi subacetatis dropped occasionally under the eyelids; it diminishes the discharge, lessens the inflammation, and is incapable of doing harm in any stage of the disease. He also praises highly the effects of a strong infusion of tobacco, two drachms of the leaves to eight ounces of water, applied directly to the affected eye.

Assalini informs us that venæsection and emollient applications to the eye were generally inefficacious, and even hurtful in many cases. After purging his patients freely, he introduced into the eye a few drops of a solution of pure potassa, or of the nitrate of silver, to which was sometimes added a small quantity of the acetate of lead. Sir P. M'Gregor states, "that local applications were found most advantageous." During the early stage he recommended one general bleeding, and a strict enforcement of the antiphlogistic regimen. Leeches were applied in large numbers near the affected organs; but of all remedies he derived the greatest benefit from the ung. hydrarg. nitrat. weakened at first with twice its quantity of lard, and applied by means of a camel-hair pencil. Sometimes the red precipitate ointment, and the quack medicine called the golden ointment, answered better than the nitrated one. Blisters applied behind the ears, or in the nape of the neck, but not near to the eye, were generally found useful. When the inflammation had somewhat subsided, he used a collyrium, composed of the nitrate of silver in water, with the greatest service. Mr. Ware, although friendly to bleeding, never trusted to the antiphlogistic treatment alone. He strongly recommended the use of the aqua camphorata of Bates' Dispensatory, and which consisted of camphor, sulphate of copper, and Armenian bole, dissolved in boiling water. Beer, the celebrated German oculist, has little faith in large depletions of blood. He employs drastic purgatives, nauseating doses of antimonials, and tepid astringent lotions, of which the best is made by dissolving one grain of corrosive sublimate in eight or ten ounces of water, to which a small quantity of vinum opii is to be added.

These authorities are perhaps sufficient to prove that the antiphlogistic treatment, however appropriate and useful it may be, in the early stages of the Egyptian purulent ophthalmia, is not in itself sufficient to arrest the progress of this formidable disease; and that the simultaneous use of powerful local applications ought never to be neglected. The same remark is applicable to the purulent ophthalmia of infants. "Of course," says Mr. Ware, "emollient applications must not be used." On the contrary, astringents and corroborants are immediately indicated, in order to restore to the vessels of the conjunctiva their original tone, and to check the morbid secretion of matter. For this purpose, no application is so beneficial as the aqua camphorata, already alluded to. Dr. Vetch speaks in high terms of the good to be derived from the undiluted liquor plumbi, and the red precipitate ointment. Mr. Guthrie, while he recommends bleeding in violent cases, trusts chiefly to the local application of the nitrate of silver, in the form either of solution or of ointment, to which a few drops of liquor plumbi have been added. It is unnecessary to adduce the opinions of other authors, as they almost uniformly agree in expressing the necessity of a vigorous local, as well as of a general treatment.

Analogy therefore leads us to employ similar means in that form of purulent ophthalmia, which is induced by the application of gonorrhœal matter of the eye. The appalling rapidity of the disease demands the most bold and determined practice on the part of the surgeon. A few hours may determine the fate of the affected eye; and we have already seen that even the most active depletion has often failed to prevent the mischief. The severe operation recommended by M. Sanson may not be always requisite; but we quite agree with the principle which directs his treatment. Let the patient be immediately bled to 16 or 20 ozs. A strong drastic should be given; and repeated doses of the tartrate of antimony to induce and keep up a state of constant nausea. These means will be sufficient to keep down all constitutional excitement; but they are not sufficient to prevent serious mischief in the affected organ. To fulfil this most necessary induction, let the surgeon, after syringing away every drop of discharge from the eye, evert the lids, and rub them freely with a stick of the nitrate of silver. A rag dipped in cold water, or a cold poultice, should then be laid over the eye. This simple, but efficacious treatment has proved most successful in our own practice, and we can therefore confidently recommend its adoption. The pain produced by the application of the caustic is generally very severe, and lasts for half an hour and upwards. The matter ought to be washed away with any mild diluent, used tepid, every two or three hours. The caustic may require to be repeated at the end of twelve hours in severe cases; in milder cases the application once in 24 hours, will be found sufficient. As the discharge abates, and becomes more thin, the use of the common astringent collyrium, of sulphate of zinc, or of alum dissolved in rose water, should be used frequently with a syringe.

In the purulent ophthalmia of infants, we have adopted a similar mode of treatment with almost uniform success. When the febrile irritation is great, a leech or two behind the ears, repeated doses of the hydrarg. cum creta, with the nitrate of potass, frequent syringing of the eyes with tepid water and milk, and the daily application of the nitrate of silver in substance, to the everted palpebræ, have, in the majority of cases, speedily induced a favorable change in the symptoms; and the cure has been completed by using a mild astringent lotion for a week or two afterwards. Under this treatment the disease is speedily, as well as effectually removed.

---

### Acute Rheumatism treated with Large Doses of the Tartrate of Antimony.

A mason, 34 years of age, was admitted into the Hospital Necker, on the 4th of October, 1833. For some days he had experienced sharp pains in the knees and wrists, with frequent shiverings and headache. The affected joints were red, and swollen, the skin hot and dry, the urine high-coloured, the pulse rather frequent. The bowels had not acted for six days.

He was ordered to be bled to 16 ozs., to be purged with sulphate of soda, and to have hot poultices applied over the painful joints. On the sixth, the pains were more severe; the elbow and shoulders had become affected; there was general restlessness, and also want of sleep. He was again bled to 16 ozs. and ordered to take "petit-lait emetise." 7th. Blood sizy. The emetic had purged the bowels several times—pains somewhat relieved. On the 11th and 12th the symptoms were much mitigated. On the 13th and 14th the pains returned with great severity—numerous leeches were applied over the affected joints. On the 16th, the disease being still very distressing, and the depletory treatment hitherto pursued, having proved ineffectual, M. Bricheteau determined to give a trial to full doses of the tartrate of antimony: 10 grains were dissolved in six ounces of water, and an ounce of poppy syrup added to it. Of this mixture, a large spoonful was to be taken every hour. 17th. The medicine had purged, but not vomited the patient. The rheumatic pains were much relieved. Medicine to be repeated with twelve grains of the antimonial; and poultices to be applied to the joints. On the 18th and 19th, the patient felt himself so much relieved that he thought he was cured. Slight numbness only was felt in the limbs. The antimonial potion, but with less of the salt, was ordered to be continued. For several days he was so well as to be able to walk out, and his appetite returned with vigour. He imprudently exposed himself to the cold damp air on the 30th, and on the following day, there was an evident relapse of all his sufferings, which were as severe as at the first attack. He lay in bed and could not move a joint, but with pain and extreme difficulty. The antimonial potion with eight grains and with some poppy syrup was immediately ordered. Fortunately the medicine produced no evacuations, either upwards or downwards; the tolerance was complete from the first dose. This was a favorable sign. In the course of two days, the relief was very striking; all pain had ceased, and the tumefaction of the joints had almost entirely subsided.

On the 10th of November, the patient was discharged cured.

*Remarks.* M. Bricheteau deduces the following two practical inferences from this case. First, that full doses of the tartrate of antimony cured the rheumatic affection, twice successively in the same patient; and secondly, that sanguineous depletion failed even to afford any very decided relief to the disease; an event which he acknowledges to be of rare occurrence, and which he thinks, was probably owing to some peculiarity "de la constitution medicale de la saison." He admits that the use of the antimonial was made at a favorable moment, "parceque la saignée avait echoué, et que c'est particulierement dans ce cas qu'il convient de recourir à la medication contre-stimulante."

*Case* 2. A man, 40 years of age, was admitted into the hospital on 17th October. For eight days previously he had suffered severe rheumatic pains in his knees, shoulders and wrists, which he was unable to move, except with acute distress. The joints were red, swollen and hot. A mixture containing eight grains of emetic tartar, and an ounce of poppy syrup was ordered to be taken in divided doses, every two, or three hours. Warm fomentations and poultices were applied to the joints.

18th. The antimonial has not caused any evacuation. The pains are less. The dose of the medicine to be increased to twelve grains.

19th. The pains are exceedingly severe; the patient cannot move in bed, but with great suffering. No nausea, or vomiting. Bowels purged thrice. The pulse has fallen to 52. The dose of the tartrate to be raised to 20 grains.

20th. No evacuation. The state of the joints not improved. To be bled to 16 ounces.

21st. Pains almost entirely removed. Blood drawn very buffy. Pulse softer and more frequent, owing doubtless to the omission of the tartrate. From this date, the amendment was progressive. Slight pains in the shoulders only, and a degree of stiffness of the joints remained. The appetite speedily became vigorous, and the patient was able to leave the hospital on the 28th of the month.

*Remarks.* In this case the antimonial was administered too soon. The bleeding ought to have preceded its use. The Italian physicians act wisely in employing these two powerful remedies simultaneously; the action of the one appearing to promote that of the other. As a general rule, one or more bleedings should precede the antimonial treatment. The influence of the medicine on the pulse was very marked. The retardation from 86 to 52 during its use, and its speedy acceleration, after its discontinuance, could be attributed to no other cause. This case also proves the perfect safety of the remedy, even when it fails to remove the disease for which it was prescribed. No disturbance of the stomach or intestines was induced.

In the third case, which occurred in a strong, plethoric, middle aged woman, and in which the usual symptoms of general acute rheumatism were well developed, the antimonial produced, on the first day, vomiting and purging. On the following day, 16th, the tolerance was quite established, and the dose was increased from eight to ten and twelve grains. 17th. Tolerance continues—pains greatly relieved—profuse diaphoresis: antimonial mixture to be continued. 18th. The patient considers herself cured, and is unwilling to take any more of her medicine, which has begun to sicken her. The swelling and pain of the joints have quite ceased —appetite keen. She remained in the hospital another week, to recover her strength, and then was discharged well.

*Remarks.* This case is interesting, from the decision and promptitude of the therapeutic effects of the antimonial, "qui a eu seul l'honneur de la cure." It is worthy of notice, that the drug exerted its usual evacuating effects as soon as the rheumatism declined, because then, no doubt, "il n'y avait plus de contre-stimulation possible, ou, si l'on veut, de rapport entre l'entité morbide, et l'action du medicament." Few cases of acute rheumatism are so speedily cured, when large bleedings have been employed, as this was; and, fortunately, the weakness which necessarily follows the depletory treatment was avoided in the present case. On the sixth day after commencing the use of the antimonial the patient was able to rise from her bed.

As for any unpleasant effects imputed to the employment of the tartrate in large doses, M. Bricheteau has not experienced them in any of the numerous cases which he has treated with it. In upwards of twenty cases of acute rheumatism, he has used it freely, and almost always with very satisfactory results.

The powerfully antiphlogistic action of the drug in pneumonia is well illustrated by the following brief details, from our author's experience.

The subject of the first case had been bled three times, blistered, &c. The disease was unchecked, and every appearance indicated an unfavourable issue, when the use of the tartrate was commenced. The patient recovered.

In the second case, one venæsection, followed by the use of the tartrate, promptly arrested the pneumonia.

In the third case, the disease had been arrested by four bleedings. A relapse took place, and was speedily cured by the antimonial treatment.

In the fourth and fifth cases, bleeding and the use of the tartrate were simultaneously resorted to, on the first day of the treatment, according to the method followed in Italy—"berceau de la medecine contre-stimulante." The use of the antimonial required to be carried to the extent of 18 grains per diem, and to be persevered in for several days, in consequence of the extreme pertinacity of the symptoms in both cases.

In the sixth case, the antimonial was first employed after bleeding—then recourse was had to bleeding alone; but the state of the patient becoming worse, the use of the antimonial was re-commenced, and the dose increased to 18 grains in the course of the 24 hours. The effect was most decided and salutary.

The quantity of the tartrate usually exhibited by M. Bricheteau did not exceed from 8 to 20 grains. He never pushed its use to the extent recommended by the Italian physicians. Whenever the resolution of the disease was once fairly established, the medicine was discontinued. "J'ai eté loin de me répentir d'avoir suivi cette marche; et le succés m'a convaincu, qu'en cette circonstance, comme en beaucoup d'autres, il ne faut jamais accabler la Nature de secours dont elle n'a pas besoin." The physician, however, need not be afraid of persisting in the use of the medicine, if the symptoms do not yield. No deleterious effects have, in M. Bricheteau's practice, ever followed the antimonial treatment. If the tolerance of the drug is not speedily induced, a few drops of laudanum, added to the mixture, will very generally enable the stomach to retain it. The formula for the mixture which he orders, is five ounces and a half of orange flower infusion, half an ounce of poppy syrup, and the prescribed quantity of the tartrate—the dose, a table-spoonful every hour, or more frequently when the symptoms are urgent.

With respect to the modus operandi of the tartrate, Laennec was of opinion that it acted, " comme excitant de l'économie animale, et du systême absorbant en particulier." Without denying to this heroic remedy the power of increasing the interstitial absorption, and of resolving in this manner a pneumonic inflammation, we are of opinion that it exerts a very marked action on the organs of secretion, and upon the entire system of the exhalants. At the same time that it eliminates and drives outwardly the excrementitial fluids separated from the mass of blood, it slackens and retards the circulation of this fluid along the sanguiferous vessels. The pulse very often falls 10, 15, or 20 beats, within the first day after the use of the tartrate has been commenced; and it was usually observed, that if, from any cause, it was discontinued for a short time, the pulse very speedily rose in frequency. It is a question open for discussion, whether or not the medicine, when the tolerance is once fairly induced, or, in other words, when no sensible evacuation follows its administration, is absorbed, and carried into the mass of circulating fluid, stimulating the secretory organs, and diminishing the excess of blood in any part which is congested or overcharged.

When the tolerance is not induced, the tartrate evidently acts as a very powerful derivative, by stimulating the stomach, intestinal canal, liver, skin, and kidneys. The effects of the large doses are, then, quite the same as those of the drug exhibited in the small doses, to which Bordeu the elder, Serane de Montpellier, &c. trusted so much in the treatment of pneumonia, as we are informed by Th. Bordeu. As to the evacuations induced by large doses of the tartrate, those from the bowels are usually more severe and obstinate than the vomitings, the lower portion of the small intestines, and the commencement of the colon, being the parts apparently most sensitive to its action. We have already stated that a few drops of laudanum, added to the solution, will generally enable the stomach and bowels to bear the use of the antimonial. When, however, this is not the case, we must discontinue its employment. The tartrate has been known to induce a congestion and infiltration of the submucous tissue of the bowels, and even a softening of the mucous coat. The lining membrane of the mouth has sometimes become inflamed, and even ulcerated, during the use of the remedy.

Pneumonia and acute rheumatism are the two phlegmasiæ which are most speedily and effectively benefited by the tartrate, given in large doses. Bleeding should generally precede its exhibition at first; but, should this be impracticable, or from any cause unadviseable, recourse may be had immediately to the use of the tartrate. Sometimes the medical constitution of the season is decidedly unfavourable to sanguineous depletion; under these circumstances, the tartrate " est un moyen precieux." The antimonial treatment will be found admirably adapted to country medical practice, where the physician cannot visit his patients so frequently as he may wish, for the purpose of watching the effects of bleedings.—" Il lui sera possible, par cette méthode, et avec le secours d'une personne intelligente, de regler le traitement d'une pneumonie, ou d'un rheumatisme, pour plusieurs jours apres avoir fait une large saignée, s'il le juge convenable." The antimonial medication is very generally well suited to old patients, whose vital powers cannot bear large or frequent depletions of blood, and in whom the mucous coat of the stomach and intestines is not so apt to be irritated by the drug as in younger subjects. The rational signs which indicate success from the use of the tartrate, are the tolerance, or absence of evacuation, being induced after the second or third dose of the medicine—the retardation of the pulse within the first twenty-four hours—moderate diaphoresis, and the feeling of greater general comfort on the part of the patient. The physical signs of an improvement speedily follow. As there are certain medical constitutions, or conditions of climate, season, &c. which contra-indicate large depletions of blood, so there are others " eminemment phlogistiques, qui interdisent l'emploi du tartre stibié." Thus it was most successfully used by me (M. Bricheteau) in 1831; while, towards the close of 1832, and at the beginning of 1833, "il nous devint impossible de l'administrer avantageusement." In the Autumn of 1833, "il nous parut utile de revenir à l'usage de ce medicament."—*Clinique Médicale, par M. Bricheteau.*

---

Scene in the French Academy.—Homœopathy.

An application having been recently made to the Minister of Public Instruction, by some homœopathists in Paris, for permission to establish a dispensary and hospital of "clinique homœopathique, pour completer l'enseignement," the Minister referred the question to the decision of the Academy of Medicine, as one of medical police, and, therefore, involving the public health. The Bureau proposed to nominate a committee, who should examine the claims of the petitioners *(general confusion.)*

M. Maingault rose, to suggest that, for the sake of impartiality, the committee should consist of as many believers as unbelievers in homœopathy *(universal uproar)*.

M. Deneux requested M. Maingault to name the members who believed in the doctrine *(laughter.)*

M. Marc did not see the propriety of having a chemist in the committee, and proposed M. Andral (the son), who had made some trials of homœopathy, in the room of M. Boullay.

M. Lodibert objected to M. Marc's proposal, and asked who could so well judge of the homœopathic pharmacopœia as a chemist.

M. Andral, senior, exclaimed, with much emotion, "I much doubt whether the Minister has any right to require a report from the Academy on an absurdity. The President should write to the Minister, and expose the cheats and juggleries of these rogues, who call themselves homœopathists. I protest against the appointment of any commission, and, therefore, move the order of the day *(loud noise—the confusion at its height)*.

M. Londe.—Write to the Minister that they have been tampering with his credulity, and that the Academy cannot condescend to have any thing to do with such charlatans, who will, no doubt, avail themselves of the very present discussion to announce, in tomorrow's paper, that the Academy were engaged with the consideration of the very important subject of homœopathy.

M. Lepelletier.—Yes! homœopathy is, indeed, a very imposture; but here we have an opportunity of exposing and abolishing it. Let us accept the challenge that is offered.

M. Keraudren suggested that an application should be made to some of the German societies, for information relative to the indigenous homœopathists *(exclamations—interruption)*.

M. Marc.—In Germany, homœopathy has fallen into the most utter contempt. A distinguished German professor assured me, the other day, that in Berlin there were only three homœopathists—one rogue and two fools *(laughter)*.

M. Breschet.—I was lately at a meeting of upwards of 600 German physicians and surgeons. Some one wished to discuss the question of homœopathy; but the proposal was rejected with such general disapprobation, that even the bare mention of a subject, which is believed only by quacks and impostors, was immediately scouted.

M. Renauldin was of opinion, that the Academy were bound to obey the request of the Minister, who had proposed for their consideration a question of medical police, and not a problem of science. Several other members took the same view; and, at length, it was agreed to appoint a committee to report.

In order that the public may be able to judge for themselves, of the credibility due to the assertions of the homœopathists, we shall extract from their own journal, the "Bibliotheque Homœopathique," some of the wonderful wonders which the infinitesimal medication has achieved within the last half-year. Dr. Peschier, secretary of the French Homœopathic Society, reports several cases of pleurisy and pneumonia, cured by a single globule of aconitum. The mere cure, however, is the least wonderful part of the history.

The extraordinary rapidity and the infallible certainty of the remedial effects, and then the confident predictions by Dr. P. of the exact moment, at which the happy change from imminent danger to complete recovery would inevitably take place, are events which, in importance and authenticity, can be matched only by the miraculous achievements of the Pope himself, in the most palmy days of Catholic ascendancy. A young lady was labouring under a most violent pleurisy, in the afternoon of ——. Dr. P. saw her at half-past 9, p.m. and ordered her " un seul globule aconit," assuring her attendants, that all the distressing symptoms would be relieved at two o'clock of the following morning, after a previous transitory aggravation. As a matter of course, the aggravation, and then the cure, took place as predicted; and the patient was quite able to resume her domestic duties next day.

But the following case is still more worthy of notice, and indeed is " le plus etonnant que Dr. Peschier a jamais rencontré." F. B. a mechanic was suddenly seized with a violent hæmoptysis. A venæsection having been practised, without any benefit, Dr. P. was summoned to the man's relief. He found his patient " crachant à pleine bouche le sang dont son vase de nuit etait à moitié plein." Availing himself (we are told) of a short interval between the very frequent attacks of the hæmoptysis, Dr. P. administered " une seule dose imperceptible de granules imbibés d'alcool aconité," and ordered him to have cold water as a drink, interdicting the employment of any other remedy, either outwardly or inwardly. Four hours afterwards he revisited him, and found that the hæmorrhage had entirely ceased, and that the other pectoral symptoms were greatly mitigated. The aconite having already " à peu prés terminé son action," the pulsatilla was substituted, and so confident was the Doctor in his auguries, that he desired the attendants to place at the bed-side two large jugs of cold water, and leave him, " parce que la nuit serait bonne." Next morning, this man, whose life had been in the most imminent danger, was able to get up and dress himself " ne sentant aucun mal, et me demandant la permission de s'aller promener." In the pride of his triumph, the doctor exclaims " certes, c'est là une des guerisons les plus rapides, les plus etonnantes dont on puisse entendre parler."

Dr. P. had asked, it seems, some Allopathic doctors, what length of time such a case, as the preceding one, would require for treatment " d'apres la methode de l'ecole," and on being told, three weeks at least, he calls on his readers to notice, that " avec homœopathie un jour avait suffi, convalescence comprise." After such a miraculous feat, it is unnecessary to allude to some minor achievements recorded by Dr. P. such as cases of epistaxis cured by a single drop of a tincture of aconite, " qui opera avec une très grande rapidité." Two cases of croup, and a case of universal tetanus were strangled at once, by the same all-powerful remedy. The most malignant fevers were almost uniformly cured by aconitum, rhus and nux, while patients treated " avec tout l'appareil de l'allopathie" were falling victims to the disease, in great numbers. The worst cases of erysipelas seldom required more than one dose of belladonna, for their entire removal. Rheumatisms, of many years standing, were charmed away by a hundredth part of a drop of rhus : pains of the bladder, by a similar dose of bryony; the agonies of nephritis, dissipated in a moment, by a spoonful of a mixture, containing one drop of nux in six ounces of water, and the most violent quinsies by a single infinitesimal dose of belladonna. Not satisfied with reducing the whole domain of pure physic to submission, the homœopathists have extended their conquests to more than one department of surgery; and, although they have not yet professed to knit broken bones, or dissolve urinary calculi, without the aid of allopathism, we are assured that contusions, however severe, may be always instantaneously relieved, and very generally at once cured, by fomenting the parts with water, in which one drop of arnica has been dissolved!

